# Unveiling the role of soil microorganisms in indicating paddy soil health via metagenomics combined with machine learning

**DOI:** 10.1093/ismeco/ycag133

**Published:** 2026-05-15

**Authors:** Yu-Ling Zheng, Yun-Shuo Guo, Xin-Yue Ren, Yi-Fei Wang, Hui-Ling Cui, Li-Mei Zhang, Long-Jun Ding, Yong-Guan Zhu

**Affiliations:** State Key Laboratory of Regional and Urban Ecology, Research Center for Eco-Environmental Sciences, Chinese Academy of Sciences, Beijing 100085, China; University of Chinese Academy of Sciences, Beijing 100049, China; State Key Laboratory of Regional and Urban Ecology, Research Center for Eco-Environmental Sciences, Chinese Academy of Sciences, Beijing 100085, China; University of Chinese Academy of Sciences, Beijing 100049, China; University of Chinese Academy of Sciences, Beijing 100049, China; State Key Laboratory of Regional and Urban Ecology, Institute of Urban Environment, Chinese Academy of Sciences, Xiamen 361021, China; State Key Laboratory of Regional and Urban Ecology, Institute of Urban Environment, Chinese Academy of Sciences, Xiamen 361021, China; Zhejiang Key Laboratory of Pollution Control for Port-Petrochemical Industry, CAS Haixi Industrial Technology Innovation Center in Beilun, Ningbo 315830, China; State Key Laboratory of Regional and Urban Ecology, Research Center for Eco-Environmental Sciences, Chinese Academy of Sciences, Beijing 100085, China; University of Chinese Academy of Sciences, Beijing 100049, China; State Key Laboratory of Regional and Urban Ecology, Research Center for Eco-Environmental Sciences, Chinese Academy of Sciences, Beijing 100085, China; University of Chinese Academy of Sciences, Beijing 100049, China; State Key Laboratory of Regional and Urban Ecology, Research Center for Eco-Environmental Sciences, Chinese Academy of Sciences, Beijing 100085, China; University of Chinese Academy of Sciences, Beijing 100049, China; State Key Laboratory of Regional and Urban Ecology, Research Center for Eco-Environmental Sciences, Chinese Academy of Sciences, Beijing 100085, China; University of Chinese Academy of Sciences, Beijing 100049, China; State Key Laboratory of Regional and Urban Ecology, Institute of Urban Environment, Chinese Academy of Sciences, Xiamen 361021, China

**Keywords:** soil health, indicator taxa, metagenomics, machine learning, paddy soils

## Abstract

The soil microbiome performs various ecological functions, making it a potentially vital component of soil health assessment; however, the indicator taxa of soil health remain unidentified. This study explored these taxa in paddy soils of the black soil region in Northeast China. First, the soil health index (SHI) was evaluated using representative physicochemical and biological parameters, revealing that approximately one-third of the soils had a low health level. A Random Forest model was then developed based on microbial species’ relative abundance to predict the SHI, achieving an *R*^2^ value greater than 0.6. Based on the SHapley Additive exPlanations values of this model, 40 microbial species were identified as potential indicator taxa of soil health, with 39 of these taxa occurring in more than 50% of the samples. Specifically, paddy soils with more abundant carbon (C)- and nitrogen (N)-fixing bacteria exhibited higher soil organic matter and total N contents, along with higher health levels. Conversely, soils rich in denitrifying bacteria exhibited lower SHI values because of increased N loss. Furthermore, C-fixing, N-fixing, and denitrifying genes showed functional relationships with the corresponding soil properties and SHI. In addition, halophilic, halotolerant, and eutrophic bacteria indicated soil health by reflecting salinity and nutrient status. The potential of these indicator taxa was validated at multidecadal and regional spatial scales. These results highlight the practical value of such indicator taxa, which elucidate the ecological processes associated with soil health and respond predictably to changes in soil health, thereby serving as rapid diagnostic tools for assessing soil health.

## Introduction

Soil health is commonly defined as the continued capacity of soil to function as a vital living ecosystem that supports plants, animals, and humans [1, 2], as appropriate to its environmental context [3]. Current soil health assessments primarily depend on physicochemical and biological properties, such as organic matter, levels of available nutrients, and enzyme activity [4–6]. However, although soil-resident microbes play a vital role in ecosystem services, they are rarely considered in the direct evaluation of soil health [7].

Soil microbial communities are particularly sensitive to environmental changes and may therefore provide valuable insights into the biological, chemical, and physical status of soils [8]. Certain soil microbes, particularly bacteria, can function as effective indicators of environmental disturbances, such as nitrogen (N) deposition, salt stress, and soil degradation [9–11]. In addition, soil microbes are involved in multiple processes that underpin soil health, including aggregate formation, suppression of soil-borne diseases, water retention, and erosion control [12, 13]. Recent advances have highlighted the vital role of soil microbiota in maintaining and evaluating soil health [14–16]. However, the taxonomic characteristics of microbial communities that shift in response to soil health changes remain unclear, thereby hindering the application of indicator taxa in soil health assessment.

Machine learning (ML) offers a powerful approach to bridge this gap by identifying indicator taxa [17]. Microbiome-based ML models have been increasingly applied in diverse contexts, including monitoring soil and groundwater pollution [18, 19], predicting crop yields [20], and diagnosing soil-borne diseases [21]. For example, ML methods leveraging bacterial taxonomy derived from 16S rRNA gene amplicon sequencing have been used to identify potential indicator taxa of soil health [22]. Moreover, metagenomics provides higher-resolution taxonomic profiling than 16S rRNA amplicon sequencing and avoids biases that may be introduced by polymerase chain reaction amplification [23, 24]. In addition, metagenomic data enable the characterization of the functional attributes of the soil microbiome, facilitating a more comprehensive understanding of the ecological roles of soil microbial communities under changing environmental conditions. Thus, integrating metagenomic sequencing with ML models has the potential to more accurately identify indicator taxa of soil health. However, a critical gap remains in current research, as high-resolution metagenomic technologies have been rarely used to precisely identify indicator taxa of paddy soil health. Furthermore, the specific ecological functions of these taxa and their spatiotemporal robustness remain poorly understood, further complicating efforts to monitor and maintain soil health in important agricultural regions.

The black soil region of Northeast China is one of the four major black soil regions in the world [25]. In recent years, intensive agricultural practices have resulted in declines in soil organic matter (SOM) and degradation of ecological functions in black soils [26]. Given that paddy rice is one of the most important crops cultivated in Northeast China, with high annual yields [27], the timely and accurate diagnosis of the health status of paddy soils in this region is of great importance. To address these challenges, we employed metagenomic sequencing combined with ML models to identify indicator taxa of paddy soil health and explore their functional attributes. The specific objectives of this study were to: (i) evaluate the soil health index (SHI) of paddy soils across this region; (ii) develop ML models to identify potential indicator taxa of paddy soil health; (iii) explore the relationships between these taxa, their associated functional genes, and key soil characteristics; and (iv) validate the generalizability of the identified taxa.

## Materials and methods

### Site description and soil sampling

**Figure 1:**
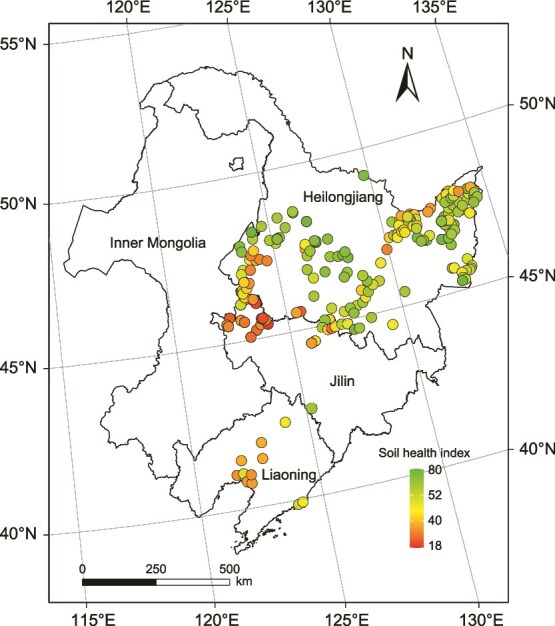
Geographical distribution of SHI of the 243 tested paddy soils in Northeast China.

Soil samples were collected from flooded paddy fields in the black soil region of Northeast China, covering 514 877 km^2^ (39.87–49.17°N; 121.68–134.48°E). Sampling was conducted before harvest each summer between June and September from 2021 to 2024, and a total of 243 samples were ultimately collected (Fig. 1). The sampling period was selected to minimize interference from anthropogenic activities (e.g. fertilizer and pesticide application). The region has a temperate monsoon climate, with a mean annual temperature (MAT) of 1°C–10°C and a mean annual precipitation (MAP) of 386–932 mm. Detailed information about this region is provided in the Supplementary Materials and Methods. The MAT and MAP data for all soil samples are presented in Table S1.

For each soil sample, five cores were independently collected from the surface layer (0–15 cm) and then thoroughly mixed to obtain a composite sample. The samples were transported to the laboratory on ice within 12 hours of collection and processed as described in the Supplementary Materials and Methods. Typical physicochemical and biological soil properties were selected because they provide information related to soil health and function, including buffering capacity, represented by pH and electrical conductivity (EC); nutrient stocks, indicated by SOM and total nitrogen (TN); the capacity to supply available nutrients required for plant and microbial growth, represented by dissolved organic carbon (DOC), dissolved total nitrogen (DTN), ammonium nitrogen (NH_4_^+^–N), nitrate nitrogen (NO_3_^−^–N), available phosphorus (AP), and available potassium (AK); and microbial nutrient cycling functions, indicated by β-glucosidase (BG), urease, and phosphatase (PHOS) activities. The analytical methods and soil properties are detailed in the Supplementary Materials and Methods and Table S1.

### Quantification of the SHI

The SHI for each sample was calculated using the mean values of all soil property scores. These averages were normalized to values between 0 and 100 based on the cumulative normal distribution (CND) and a scoring function. The CND value was first calculated using Equation (1) [4]:


(1) \begin{equation*} \mathrm{CND}(x)=\frac{1}{\sigma \sqrt{2\pi }}{\int}_{-\infty}^{+\infty }e\frac{-{\left(x-\mu \right)}^2}{2{\sigma}^2} dx \end{equation*}

where *x*, *μ*, and *σ* represent the measured value, mean value, and standard deviation of each soil property, respectively. Three types of scoring function types were then applied to evaluate the soil properties. (i) A “more is better” function was applied to properties positively associated with soil health, including SOM, TN, DOC, DTN, NH_4_^+^–N, NO_3_^–^–N, AP, AK, BG, urease, and PHOS [28–30]. The scores for these properties were calculated as: score = 100 × CND. (ii) A “less is better” function was applied to EC, as this property is typically negatively correlated with soil health [31]. The scoring function was calculated as: score = 100 × (1 − CND). (iii) An “optimum” function was applied to soil pH within the ideal range. Specifically, pH values between 6.3 and 7.2 were considered optimal (score = 100) for nutrient availability, plant growth, and microbial activity [4]. A score of 0 was assigned for pH ≤ 5.4 or pH ≥ 7.7, with intermediate values obtained through linear interpolation between these limits and the optimal range [4]. The scoring functions are summarized in Table 1. Subsequently, the calculated SHI values for all samples were classified into low, medium, and high levels according to a previously reported methodology with minor modifications [32]. The specific grading criteria are provided in the Supplementary Materials and Methods and Table S2.

**Table 1:** Scoring functions for soil properties.

Soil function	Related soil property^1^	Type of scoring	Scoring function^2^	Reference
Buffering capacity	EC (μS cm^−1^)	Less is better	Score = 100 × (1-CND)	[31]
Nutrient stocks	SOM (g kg^−1^)	More is better	Score = 100 × CND	[28]
	TN (g kg^−1^)			[28]
Available nutrient provision	DOC (mg kg^−1^)	More is better	Score = 100 × CND	[28]
	DTN (mg kg^−1^)			[29]
	NH_4_^+^-N (mg kg^−1^)			[30]
	NO_3_^–^–N (mg kg^−1^)			[30]
	AP (mg kg^−1^)			[28]
	AK (mg kg^−1^)			[28]
Microbial nutrient cycles	BG (nmol g^−1^ h^−1^)	More is better	Score = 100 × CND	[28]
	Urease (U g^−1^)			[28]
	PHOS (nmol g^−1^ h^−1^)			[28]

^1^EC: electrical conductivity; SOM: soil organic matter; TN: total nitrogen; DOC: dissolved organic carbon; DTN: dissolved total nitrogen; NH_4_^+^-N: ammonium nitrogen; NO_3_^–^–N: nitrate nitrogen; AP: available phosphorus; AK: available potassium; BG: β-glucosidase; and PHOS: phosphatase.

^2^CND: cumulative normal distribution.

### Soil DNA extraction and metagenomic sequencing

Soil DNA was extracted using the FastDNA Spin Kit for Soil (MP Biomedicals, CA, USA) according to the manufacturer’s instructions. The extracted DNA was sequenced on a DNBSEQ-T7 platform (MGI Tech, Guangdong, China) using a 150-base pair (bp) paired-end sequencing strategy. All raw data were filtered using the SOAPnuke (v1.5.2) tool [33], which produced an average of 16 GB of clean data per sample. The methods used for the subsequent assembly of contigs, prediction of open reading frames, construction of a non-redundant gene catalog, and calculation of gene abundance are provided in the Supplementary Materials and Methods. For taxonomic and functional annotation, the DIAMOND (v2.1.11) tool was used to compare the non-redundant gene set against the National Center for Biotechnology Information non-redundant (NCBI-nr) and Kyoto Encyclopedia of Genes and Genomes (KEGG) databases [34, 35].

### ML model development

Random Forest (RF), Support Vector Regression (SVR), and eXtreme Gradient Boosting (XGBoost) algorithms were adopted to develop ML models for identifying potential indicator taxa of paddy soil health. These algorithms were selected because they can handle high-dimensional datasets and provide robust interpretability, which helps identify the most critical variables within the dataset [36]. The ML models used relative species-level metagenomic abundance profiles as the input data and the SHI value as the target variable. The final dataset comprising these variables was divided into training (80%) and testing (20%) sets. Hyperparameters were optimized using the GridSearchCV function [37], and model performance was evaluated based on the coefficient of determination (*R*^2^), root mean square error (*RMSE*), and mean absolute error (*MAE*) [38]. All ML models were implemented using the Scikit-learn library in Python 3.12 (https://pypi.org/project/scikit-learn/). Detailed descriptions of the ML algorithms used are provided in the Supplementary Materials and Methods.

### Identification of potential indicator taxa of paddy soil health

SHapley Additive exPlanations (SHAP) analysis was employed to determine the top 40 most important species for predicting the SHI [39]. Furthermore, to determine whether these species alone could accurately predict the SHI, the training and testing datasets described above were used to reconstruct the RF model. If the reconstructed model maintained strong predictive performance, the 40 species were considered capable of assessing soil health and were therefore identified as potential indicator taxa of paddy soil health. The SHAP values were calculated using the SHAP library (v0.46.0) in Python.

### External validation of potential indicator taxa

To further validate the general applicability of the RF model constructed based on the identified 40 potential indicator taxa, external datasets comprising metagenomic sequences and soil properties from two independent studies were used. The first dataset included bare soil (considered pristine) and a chronosequence of paddy soils with increasing durations of cultivation (2, 4, 6, 8, 11, 12, 20, and 23 years after reclamation; n = 63) [40]. The second dataset consisted of samples collected across the typical Mollisol region of Northeast China (n = 120) [41]. Although these external datasets were geographically distinct from our sampling locations, they shared the same black soil regional context. The 40 potential indicator taxa identified in our study were used as input variables to predict the SHI values for these external datasets. The predicted SHI values were then compared with the actual SHI values reported in the respective studies to evaluate whether the indicator taxa could serve as reliable diagnostic tools for paddy soil health in black soil regions.

### Statistical analyses

Dissimilarities in the soil microbial community structure at the species level and functional structure at the KEGG Orthology level across the tested paddy soils were visualized using non-metric multidimensional scaling (NMDS) based on Bray–Curtis distances. NMDS was performed using the vegan package (version 2.6-4) in R [42]. The statistical significance of differences in the predicted SHI values between bare and paddy-cultivated soils was determined using an independent *t-*test implemented with the *t.test* function in R [43]. All analyses were conducted in the R environment (version 4.2.1; https://cran.r-project.org/), and ArcGIS V10.2 software was used to map the SHI [44].

## Results

### Characterization of the SHI

The spatial variations in the SHI of the tested paddy soils were determined based on the examined soil properties (Fig. 1). Specifically, 30% of the paddy soils were classified as having a low health level (SHI < 40), while 35% were classified as having a medium health level (40 ≤ SHI < 52). The former were primarily located in the northwest and southwest regions of Jilin and Heilongjiang Provinces, respectively. The remaining 35% of the tested paddy soils were categorized as having a high health level (SHI ≥ 52), which were largely concentrated in the central, southern, northern, and eastern regions of Heilongjiang Province. Spearman correlation analysis further indicated that the SHI was significantly and positively correlated with MAP but negatively correlated with MAT (Fig. S1). MAP was also positively correlated with most soil properties related to nutrient stocks (SOM and TN), available nutrient supply (NO_3_^−^–N, DTN, AK, and NH_4_^+^–N), and microbial enzyme activity (PHOS and BG), whereas MAT exhibited the opposite trend.

### Soil microbial community and functional structure

**Figure 2:**
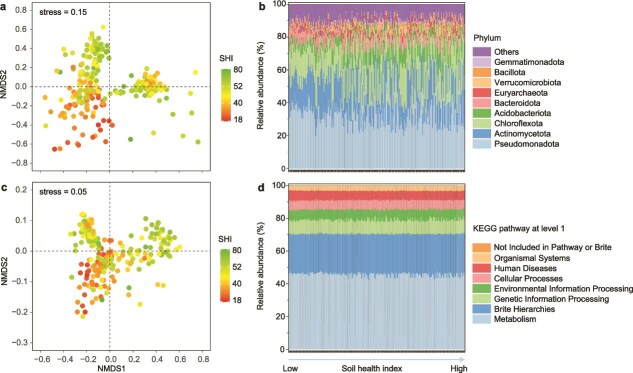
(a, c) NMDS analysis based on the Bray-Curtis distance showing the distribution of (a) microbial community structure and (c) microbial functional structure for all tested paddy soils. (b) Composition of microbiome at the phylum level in soil samples ranked by SHI. “Others” refers to the taxa with a medium proportion of less than 1% in all soil samples. (d) Profile of KEGG pathway at level 1 for all tested paddy soils. KEGG: Kyoto Encyclopedia of genes and genomes.

The results of the NMDS analysis revealed that microbial community profiles across paddy soil samples were distributed along the NMDS1 and NMDS2 axes following the SHI gradient (Fig. 2a). Regarding microbial community composition, Pseudomonadota was the most abundant phylum across all samples, with a median relative abundance of 34.18%, followed by Actinomycetota (18.36%), Chloroflexota (18.16%), and Acidobacteriota (6.59%; Fig. 2b). The top 10 most abundant genera generally belonged to Pseudomonadota and Actinomycetota, with the genus Bradyrhizobium (affiliated with the phylum Pseudomonadota) being the most abundant taxon across samples, with a median relative abundance of 2.29% (Fig. S2A). The microbial functional structures across samples were also distributed along the NMDS1 and NMDS2 axes following the SHI gradient (Fig. 2c). At level 1 of the KEGG functional annotation, genes were primarily associated with metabolism, BRITE hierarchies, genetic information processing, environmental information processing, cellular processes, human diseases, and organismal systems. The relative abundance of genes associated with metabolism was the highest, ranging from 42.46% to 48.08% (Fig. 2d). Furthermore, transporters within the BRITE hierarchies were identified as the most abundant functional category at KEGG level 3, representing 3.83% of the annotated genes (Fig. S2B).

### Development of the ML model and identification of potential indicator taxa

In this study, three ML models—RF, SVR, and XGBoost—were developed by comparing the predicted and measured SHI values using overall species-level taxonomic data as explanatory variables. The RF model exhibited a testing *R*^2^ value of 0.65, with corresponding *RMSE* and *MAE* values of 7.71 and 6.23, respectively (Table S3). In comparison, the SVR model showed a lower testing *R*^2^ value of 0.62 and higher *RMSE* (8.02) and *MAE* (6.51) values. Similarly, the XGBoost model exhibited a lower testing *R*^2^ value of 0.60 and higher *RMSE* (8.21) and *MAE* (6.60) values (Table S3). These results indicate that the RF model with optimally tuned hyperparameters performed better than the SVR and XGBoost models. Therefore, the RF model was subsequently used to identify potential indicator taxa of paddy soil health.

**Figure 3:**
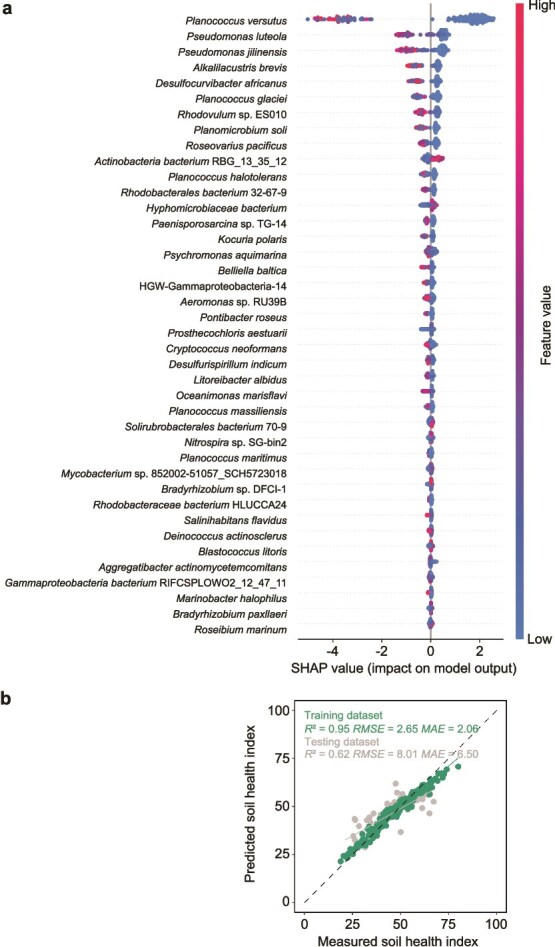
(a) SHAP summary plot for the 40 most important features predicting soil health index (SHI) based on the random forest model. A positive SHAP value reflects that the feature has a positive influence on the predicted SHI, while a negative value reflects a negative influence. (b) Performance of the reconstructed RF model to predict SHI for the examined paddy soils. Dashed black line indicates the perfect prediction with the predicted values being equaled to the measured values. The green dots and lines represent the training dataset, and the grey dots and lines represent the testing dataset.

Based on the SHAP values of the RF model, 40 microbial species (including 39 bacteria and one fungus) that were most important for predicting the SHI were identified (Fig. 3a). Among these, *Planococcus versutus* was identified as the most important predictive taxon, followed by *Pseudomonas luteola* and *Pseudomonas jilinensis*. *Solirubrobacterales bacterium* 70–9 exhibited the highest mean relative abundance and was present in all tested samples. Furthermore, *Hyphomicrobiaceae bacterium*, *Kocuria polaris*, *Prosthecochloris aestuarii*, *Deinococcus actinosclerus*, *Blastococcus litoris*, *Bradyrhizobium paxllaeri*, and *Roseibium marinum* were consistently detected across all samples. All species, except *Planococcus halotolerans*, exhibited an occurrence frequency greater than 50% (Fig. S3). Spearman correlation analysis revealed that 37 species were significantly (*P* < 0.05) associated with soil health (Fig. S4). Specifically, 28 species, most of which belonged to the orders Bacillales, Cytophagales, and Desulfovibrionales, were significantly negatively correlated with the SHI. In contrast, nine species, predominantly from the orders Solirubrobacterales, Hyphomicrobiales, and Geodermatophilales, exhibited significant positive correlations with the SHI.

To evaluate the SHI prediction ability of these species, a new RF model was constructed using their relative abundances as explanatory variables. The testing *R*^2^ value of this model was 0.62 (Fig. 3b), which was comparable to that obtained using the overall species-level taxonomic data (*R*^2^ = 0.65). This result suggests that these 40 species can accurately predict the paddy soil SHI and can therefore serve as potential indicator taxa of paddy soil health.

### Relationships between potential indicator taxa, functional genes, and soil characteristics

**Figure 4:**
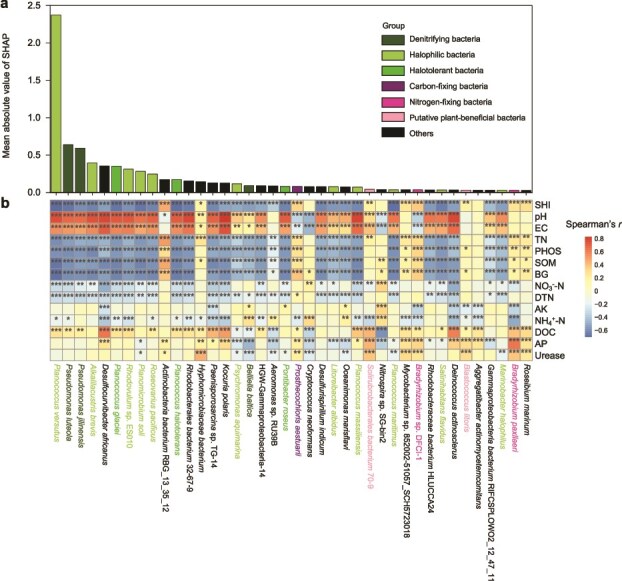
(a) SHAP bar plot for the 40 most important features predicting SHI based on the random forest model. These features are categorized into seven functional groups based on previous literature, detailed information is shown in Table S4. (b) Heatmap plot of Spearman correlation analysis between the relative abundance of indicator taxa and soil health index (SHI), as well as various soil properties. The color denotes the Spearman’s correlation coefficient. Significance level was set as ^***^ *P* < 0.001, ^**^ *P* < 0.01, and ^*^ *P* < 0.05. EC: electrical conductivity; TN: total nitrogen; PHOS: phosphatase; SOM: soil organic matter; BG: β-glucosidase; NO_3_^−^-N: nitrate nitrogen; DTN: dissolved total nitrogen; AK: available potassium; NH_4_^+^-N: ammonium nitrogen; DOC: dissolved organic carbon; and AP: available phosphorus.

The 40 identified taxa were further categorized into seven functional groups based on their primary ecological roles: denitrifying bacteria, halophilic bacteria, halotolerant bacteria, carbon (C)-fixing bacteria, N-fixing bacteria, putative plant-beneficial bacteria, and others (Fig. 4a and Table S4). Spearman correlation analysis suggested that all taxa within the groups of denitrifying bacteria (e.g. *P. luteola* and *Pseudomonas jilinensis*), halophilic bacteria (e.g. *Planococcus versutus* and *Alkalilacustris brevis*), and halotolerant bacteria (e.g. *Planococcus glaciei* and *Planococcus halotolerans*) were significantly and negatively correlated with the SHI (Fig. 4b), as well as with several soil properties related to nutrient stocks (e.g. SOM and TN), available nutrient supply (e.g. NO_3_^−^–N), and enzyme activity (e.g. PHOS and BG). However, these taxa exhibited significant positive correlations with soil pH and EC. In contrast, taxa grouped as C-fixing bacteria (e.g. *P. aestuarii*), N-fixing bacteria (e.g. *Bradyrhizobium* sp. DFCI-1 and *Bradyrhizobium paxllaeri*), and putative plant-beneficial bacteria (e.g. *Blastococcus litoris* and *Solirubrobacterales bacterium* 70–9) demonstrated significant positive correlations with the SHI and with soil properties related to nutrient stocks (e.g. AP, SOM, and TN) (Fig. 4b). Among the putative plant-beneficial bacteria identified in this study, *Blastococcus litoris* harbored the genes *glnA* and *argH*, whereas *Solirubrobacterales bacterium* 70–9 harbored the genes *glnA*, *argH*, *gabD*, and *ppa* (Table S5). In addition, among the potential indicator taxa grouped into “Others”, *Desulfocurvibacter africanus*, *K. polaris*, and *Desulfurispirillum indicum*, which harbored denitrifying genes (e.g. *narI*, *nirK*, and *napA*), were negatively correlated with the SHI, whereas *Hyphomicrobiaceae bacterium*, which harbored the alanine–glyoxylate aminotransferase gene (*AGXT*) gene, was positively correlated with the SHI (Fig. 4b and Table S5).

The relationships between the corresponding functional genes (including denitrifying genes, salt-tolerant genes, C-fixing genes, and N-fixing genes; Table S6) and the SHI were further explored. Consistent with the functional groups of the potential indicator taxa, denitrifying genes and salt-tolerant genes were both significantly negatively correlated with the SHI (Fig. S5A and B). Notably, denitrifying genes were also negatively associated with soil NO_3_^–^–N and TN levels, whereas denitrifying genes and salt-tolerant genes were positively correlated with soil pH and EC, respectively (Fig. S6A–D). In contrast, C-fixing and N-fixing genes were significantly and positively correlated with the SHI (Fig. S5C and D). Moreover, C-fixing and N-fixing genes were positively associated with SOM and TN contents, respectively (Fig. S6E and F).

### External validation of potential indicator taxa

**Figure 5:**
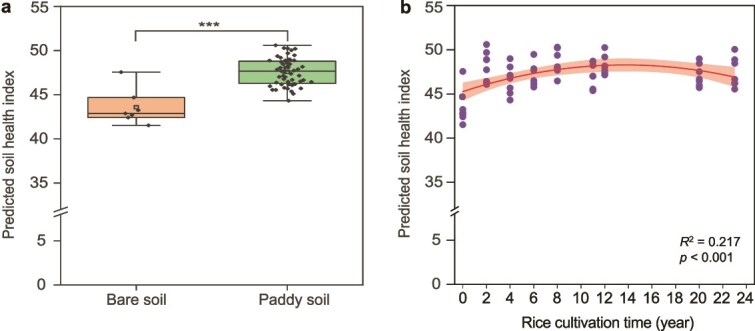
(a) Comparison of soil health index (SHI) predicted by a random forest model based on the 40 potential indicator taxa between bare soil and a chronosequence of paddy soils. (b) Dynamic changes of predicted SHI in a chronosequence of paddy soils along with increasing rice cultivation time since reclamation from bare soil. Significance level was set as ^***^ *P* < 0.001.

To assess the generalizability of the reconstructed RF model, it was applied to two independent external datasets. In the first validation study, the predicted SHI of bare soil was 43.96 ± 2.00, whereas that of soils under paddy cultivation for 2–23 years was significantly higher, at 47.57 ± 1.52 (*P* < 0.001) (Fig. 5a). The model demonstrated acceptable accuracy in predicting SHI, with an *R*^2^ of 0.312 (*P* < 0.001) (Fig. S7A). Specifically, the predicted SHI followed a temporal pattern characterized by an initial increase followed by a decline with prolonged rice cultivation; optimal soil health levels were observed after 11–16 years of continuous cultivation following reclamation from bare soil (Fig. 5b). This trend was consistent with the temporal dynamics of soil health previously reported for this region [40]. In the second validation study, the RF model also demonstrated strong predictive performance for the SHI across the typical Mollisol region of Northeast China, achieving an *R*^2^ of 0.605 (*P* < 0.001) (Fig. S7B). Collectively, these results further demonstrate the reliability and robustness of the constructed RF model.

## Discussion

In this study, we assessed soil health based on typical soil properties associated with various ecosystem services, including buffering capacity, nutrient stocks, nutrient availability, and microbial-driven nutrient cycling. Soil health was evaluated using the Cornell Comprehensive Assessment of Soil Health protocol, a standardized framework widely used for agricultural soil assessment [45]. Based on this protocol, we found that nearly one-third of the examined paddy soils had a low health level. These soils were primarily distributed in the northwest and southwest regions of Jilin and Heilongjiang Provinces, respectively, particularly within the Songnen Plain. This pattern could be explained by the frequent occurrence of soil salinization and alkalization [46, 47]. Soil salinity and alkalinity reduce nutrient availability and microbial activity, as reflected by decreased soil enzymatic activities, thereby leading to declines in soil health levels [48, 49]. In addition, these conditions can inhibit rice growth and reduce yield [50]. Therefore, improving saline–alkaline soils would likely enhance the health status of paddy soils in the Songnen Plain of Northeast China. In contrast, paddy soils located in the central, southern, northern, and eastern regions of Heilongjiang Province exhibited higher health levels than those in the other sampled regions. This variability may be attributed to the relatively high MAP and low MAT in these regions, which are conducive to the accumulation of SOM and TN, thereby improving soil health levels (Fig. S1) [51]. These geographical patterns of soil health distribution are consistent with findings from a previous study reporting that the quality of cultivated lands in the northwest of Jilin Province and the southwest of Heilongjiang Province was lower than that in other regions of these two provinces and Liaoning Province [52]. Moreover, a similar spatial pattern was observed in the soil productivity index of the typical black soil subregion of Northeast China [53], supporting our SHI results from an alternative perspective and further indicating the reliability of the paddy soil health classifications obtained. It is important to note that our study area is located within a temperate monsoon climate zone, which differs considerably from tropical or arid climates. Therefore, soil health assessments should be adapted to account for regional climatic conditions to enable accurate and meaningful comparisons across different environments.

To identify the potential indicator taxa of paddy soil health in the black soil region of Northeast China, two Random Forest (RF) models were developed. The first RF model was constructed using the relative abundances of all species, whereas the second RF model focused on the relative abundances of the top 40 species identified as the most important predictors of SHI. Compared with the first model, the second model offered several advantages. It simplified the model architecture, reduced computational complexity, and thereby enhanced practical applicability. In addition, by focusing on key indicator taxa, the second model enabled validation of their predictive capacity for soil health, including applications to geographically distinct samples. This approach therefore provides a scientific foundation for the development of future molecular diagnostic tools for soil health assessment. Consequently, constructing the second model was essential, as it improved computational efficiency while also demonstrating the model’s generalizability to external soil samples. Among the 40 potential indicator taxa of paddy soil health identified in this study, denitrifying, halophilic, and halotolerant bacteria exhibited significant negative correlations with the SHI in terms of their ecological functions (Fig. 4). Denitrification is a well-documented pathway of N loss in paddy soils [54], and a higher abundance of denitrifying bacteria is associated with greater N loss [55]. This pattern was corroborated by our findings, in which all denitrifying bacterial populations identified as potential indicator taxa showed strong negative correlations with the SHI, NO_3_^–^–N, and TN levels, as well as positive correlations with soil pH (Fig. 4). Consistently, annotated denitrifying genes (e.g. *nirK* and *nirS*) also exhibited negative correlations with the SHI, NO_3_^–^–N, and TN levels, along with positive correlations with pH (Figs. S5A, S6A–C). Soil pH may serve as a key driver of denitrification. Previous studies have demonstrated that denitrification rates in neutral and alkaline soils can be 2.6–16.6 times higher than those in acidic soils [56]. Therefore, the negative correlations observed between denitrifying genes and NO_3_^–^–N or TN in this study may be driven by soil pH. Higher pH conditions favor greater abundances of denitrifying bacteria and associated genes, thereby enhancing N consumption and reducing residual N in the soil. Considering that NO_3_^–^–N and TN positively contribute to soil health and were assigned the “more is better” scoring function in SHI quantification [28, 30], paddy soils harboring greater abundances of denitrifying bacterial populations identified as potential indicator taxa (e.g. *P. luteola* and *Pseudomonas jilinensis*) are likely to exhibit lower health levels and reduced SHI values. Potential indicator taxa grouped as halophilic bacteria (e.g. *Planococcus versutus* and *Alkalilacustris brevis*) exhibited significant positive associations with soil EC (Fig. 4). Halophilic bacteria are known to thrive in high-salinity environments [57]. Therefore, their increased abundance reflects elevated soil salinity, as indicated by EC. Because higher salinity adversely affects paddy soil health, the proliferation of halophilic bacteria is associated with poorer soil health. Furthermore, potential indicator taxa grouped as halotolerant bacteria, such as *Planococcus glaciei* and *Planococcus halotolerans*, also displayed significant positive correlations with EC (Fig. 4), although they can readily grow under non-saline conditions [57–59]. Similarly, salt-tolerance genes harbored by halophilic and halotolerant microbes showed positive correlations with soil EC but negative correlations with SHI (Figs. S5B and S6D). These findings suggest that, as with halophilic bacteria, elevated abundances of certain halotolerant bacterial populations may indicate increasing soil salinity and consequently lower health levels of paddy soils.

In contrast, the potential indicator taxa grouped as C-fixing, N-fixing, and putative plant-beneficial bacteria were found to be significantly and positively correlated with the SHI (Fig. 4). Among them, the C-fixing bacterial species *P. aestuarii*, which can convert CO_2_ into organic matter via photosynthesis, was identified as one of the most important indicator taxa based on its SHAP value [60]. Therefore, a higher abundance of *P. aestuarii* may indicate elevated SOM levels, a hypothesis supported by the significant positive correlation between this species and SOM content (Fig. 4b). Given that SOM positively influences paddy soil health and that the “more is better” scoring function was applied in SHI quantification [61], the positive association between *P. aestuarii* and the SHI is reasonable. This finding suggests that paddy soils harboring more abundant C-fixing bacterial populations identified as potential indicator taxa tend to exhibit better health levels with higher SHI values. In addition, the strong positive correlations between potential indicator taxa classified as N-fixing bacteria (e.g. *Bradyrhizobium* sp. DFCI-1 and *Bradyrhizobium paxllaeri*) and the SHI can be explained by their capacity to fix atmospheric dinitrogen [62]. This process may increase soil total N levels, thereby positively influencing paddy soil health (Fig. 4) [63–65]. Consistent with the abundance patterns of these C-fixing/N-fixing bacteria, the annotated C-fixing/N-fixing genes (e.g. *korA*/*nifA*) also exhibited positive associations with the SHI and with SOM/TN contents (Figs. S5C, D, and  S6E, F). These findings further highlight the pivotal roles of C-fixing and N-fixing microbes in indicating paddy soil health. Interestingly, all potential indicator taxa grouped as putative plant-beneficial bacteria also exhibited significant positive correlations with the SHI, although the mechanisms underlying their contributions to paddy soil health remain unclear. Contig-based functional annotation revealed that the putative plant-beneficial bacteria *Blastococcus litoris* and *Solirubrobacterales bacterium* 70–9 harbor the arginine biosynthesis gene *argH*, which is associated with pathogen suppression and plant growth promotion [66]. Moreover, *Solirubrobacterales bacterium* 70–9 harbors the gamma-aminobutyric acid biosynthesis gene *gabD*, which promotes plant growth through the suppression of plant pathogens [67]. Both taxa also possess the glutamine synthetase gene *glnA*, a key enzyme involved in N assimilation that enhances soil N retention and contributes to improved soil health [68]. In addition, *Solirubrobacterales bacterium* 70–9 harbors the inorganic phosphorus solubilization gene *ppa*, which can increase the bioavailability of phosphorus in soil and thereby contribute to higher soil health levels [69]. Collectively, these genomic features suggest the multifaceted functional roles of the putative plant-beneficial bacteria group in supporting soil health.

In addition, among the potential indicator taxa grouped into “Others”, whose ecological roles in soil health remain largely uncharacterized, several eutrophic bacteria, including *Hyphomicrobiaceae bacterium* and *Roseibium marinum*, showed strong positive correlations with the SHI (Fig. 4) [70, 71]. As these taxa preferentially inhabit nutrient-rich environments with high C and N levels [70], paddy soils with elevated abundances of these taxa may indicate higher SOM and TN contents. Therefore, soils harboring higher abundances of eutrophic bacteria identified as potential indicator taxa are likely to occur in environments with improved soil health and higher SHI values. Contig-based functional profiling further revealed that *Hyphomicrobiaceae bacterium* harbors the *AGXT* gene, an enzyme implicated in enhancing plant antioxidant capacity and improving host stress resilience [72]. In contrast, other members of the “Others” group, such as *Desulfocurvibacter africanus*, *K. polaris*, and *D. indicum*, harbor denitrifying genes, including *narI*, *nirK*, and *napA*, suggesting their potential involvement in N loss via denitrification, which may negatively affect soil health.

Notably, the identified 40 potential indicator taxa were applied to diagnose the health status of a chronosequence of paddy soils located in the black soil region of Northeast China. The results demonstrated that these taxa could effectively reflect changes in soil health across a temporal scale spanning several decades. Moreover, their diagnostic capacity was confirmed across a broad geographic scale within the typical Mollisol region. Such external validation further highlights the potential of soil microbial populations, particularly those associated with nutrient cycling, to serve as reliable indicator taxa for paddy soil health in black soil regions, even across multi-decadal and regional spatial scales. However, potential indicator taxa associated with nutrient cycling, including denitrifying, C-fixing, and N-fixing bacteria, may influence soil health by regulating nutrient dynamics such as nutrient accumulation and loss. Other potential indicator taxa, including halophilic and halotolerant bacteria as well as eutrophic taxa, may respond predictably to environmental changes associated with soil health status. For example, their abundances may increase under conditions of elevated soil salinity or high C and N levels. These taxa could therefore be incorporated into soil health assessment systems to provide additional biologically relevant information. It is important to emphasize that the primary objective of this study was to comprehensively characterize the relationships between soil microorganisms and soil health at the regional scale. To achieve this goal, composite soil sampling was adopted as a resource-efficient strategy [73]. Although the absence of biological replicates in composite sampling may limit the resolution of certain diversity metrics derived from sequencing, particularly beta diversity and the relative abundance of taxa, such limitations do not compromise the robustness or scientific validity of our regional-scale inferences. Numerous previous studies have similarly employed composite soil samples without biological replication for high-throughput sequencing analyses at regional scales [74–76].

Overall, the findings of this study indicate that approximately one-third of the tested paddy soils in the black soil region of Northeast China exhibit low health levels. These soils were primarily distributed in the Songnen Plain, where soil salinization and alkalization are prevalent. A total of 40 microbial species were identified as potential indicator taxa of paddy soil health based on SHAP values derived from the RF model. Among these taxa, those associated with nutrient cycling (particularly C and N processes) played critical roles in indicating soil health, as they directly regulate nutrient turnover. Paddy soils with better health levels harbored more abundant bacterial populations and functional genes associated with C and N fixation. In contrast, soils with lower health levels showed higher abundances of bacterial populations and functional genes involved in N loss processes such as denitrification. In addition, several other indicator taxa, including halophilic, halotolerant, and eutrophic bacteria, may reliably indicate paddy soil health because they directly reflect soil salinity and nutrient conditions. These findings provide strong support for incorporating microbial indicator taxa into soil health assessment systems, as these taxa both regulate soil ecosystem processes and respond predictably to changes in soil health status. Given that microbial indicator taxa provide functional insights into soil ecosystem services and are sensitive to global environmental changes, monitoring their dynamics may facilitate the rapid diagnosis of soil health under climate change scenarios. Such monitoring could enable soil managers to track dynamic changes in soil health over time and develop appropriate soil management strategies.

## Supplementary Material

Supplemental_Material_ycag133

Supplemental_Material_Table_S1_ycag133

## Data Availability

Metagenomic sequencing data have been deposited at National Center for Biotechnology Information (NCBI) Sequence Read Archive (SRA) with the BioProject accession number PRJNA1057541, PRJNA924737, and PRJNA1121416. Scripts for statistical analyses are available on GitHub at https://github.com/YuLingZheng-YL/Soil_health.

## References

[1] Lehmann J, Bossio DA, Kögel-Knabner I et al. The concept and future prospects of soil health. *Nat Rev Earth Environ* 2020;1:544–53. 10.1038/s43017-020-0080-8 33015639 PMC7116140

[2] Banerjee S, van der Heijden MGA. Soil microbiomes and one health. *Nat Rev Microbiol* 2023;21:6–20. 10.1038/s41579-022-00779-w 35999468

[3] Wills S, Williams C, Seybold C et al. Using soil survey to assess and predict soil condition and change. In: Field D.J., Morgan C.L.S., McBratney A.B. (eds.), Global Soil Security. Cham: Springer, 2017, 123–35.

[4] Fine AK, van Es HM, Schindelbeck RR. Statistics, scoring functions, and regional analysis of a comprehensive soil health database. *Soil Sci Soc Am J* 2017;81:589–601. 10.2136/sssaj2016.09.0286

[5] Purakayastha TJ, Pathak H, Kumari S et al. Soil health card development for efficient soil management in Haryana, India. *Soil & Tillage Research* 2019;191:294–305. 10.1016/j.still.2018.12.024

[6] Wei L, Li YH, Zhu ZK et al. Soil health evaluation approaches along a reclamation consequence in Hangzhou Bay, China. *Agric Ecosyst Environ* 2022;337:108045. 10.1016/j.agee.2022.108045

[7] Yang T, Lupwayi N, Marc S et al. Anthropogenic drivers of soil microbial communities and impacts on soil biological functions in agroecosystems. *Global Ecology and Conservation* 2021;27:e01521. 10.1016/j.gecco.2021.e01521

[8] König S, Vogel HJ, Harms H et al. Physical, chemical and biological effects on soil bacterial dynamics in microscale models. *Front Ecol Evol* 2020;8:53. 10.3389/fevo.2020.00053

[9] He WY, Zhang MM, Jin GZ et al. Effects of nitrogen deposition on nitrogen-mineralizing enzyme activity and soil microbial community structure in a Korean pine plantation. *Microb Ecol* 2021;81:410–24. 10.1007/s00248-020-01595-6 32894355

[10] Dong Y, Chen RR, Graham EB et al. Eco-evolutionary strategies for relieving carbon limitation under salt stress differ across microbial clades. *Nature* *Communications* 2024;15:6013. 10.1038/s41467-024-50368-z PMC11255312 39019914

[11] Coban O, De Deyn GB, van der Ploeg M. Soil microbiota as game-changers in restoration of degraded lands. *Science* 2022;375:abe0725. 10.1126/science.abe0725 35239372

[12] Philippot L, Chenu C, Kappler A et al. The interplay between microbial communities and soil properties. *Nat Rev Microbiol* 2024;22:226–39. 10.1038/s41579-023-00980-5 37863969

[13] Wen T, Ding ZX, Thomashow LS et al. Deciphering the mechanism of fungal pathogen-induced disease-suppressive soil. *New Phytol* 2023;238:2634–50. 10.1111/nph.18886 36932631

[14] Xu JM, Ren CC, Zhang XM et al. Soil health contributes to variations in crop production and nitrogen use efficiency. *Nat Food* 2025;6:597–609. 10.1038/s43016-025-01155-6 40234681

[15] Jiang F, Du WC, van Groenigen KJ et al. Long-term elevated CO_2_ improves soil health and rice yields in paddy fields. *Adv Sci* 2026;13:e03190. 10.1002/advs.202503190 PMC12931181 41355054

[16] Zhang YX, Gilbert JA, Liu X et al. SynCom-mediated herbicide degradation activates microbial carbon metabolism in soils. *iMeta* 2025;4:e70058. 10.1002/imt2.70058 41112045 PMC12527992

[17] Medina R, Kutuzova S, Nielsen KN et al. Machine learning and deep learning applications in microbiome research. *ISME communications* 2022;2:98. 10.1038/s43705-022-00182-9 37938690 PMC9723725

[18] Zhang XD, Long T, Deng SP et al. Machine learning modeling based on microbial community for prediction of natural attenuation in groundwater. *Environ Sci Technol* 2023;57:21212–23. 10.1021/acs.est.3c05667 38064381

[19] Zhang Q, Zhang ZY, Lu T et al. Gammaproteobacteria, a core taxon in the guts of soil fauna, are potential responders to environmental concentrations of soil pollutants. *Microbiome* 2021;9:196. 10.1186/s40168-021-01150-6 34593032 PMC8485531

[20] Chang HX, Haudenshield JS, Bowen CR et al. Metagenome-wide association study and machine learning prediction of bulk soil microbiome and crop productivity. *Front Microbiol* 2017;8:519. 10.3389/fmicb.2017.00519 28421041 PMC5378059

[21] Yuan J, Wen T, Zhang H et al. Predicting disease occurrence with high accuracy based on soil macroecological patterns of Fusarium wilt. *ISME J* 2020;14:2936–50. 10.1038/s41396-020-0720-5 32681158 PMC7784920

[22] Wilhelm RC, van Es HM, Buckley DH. Predicting measures of soil health using the microbiome and supervised machine learning. *Soil Biol Biochem* 2022;164:108472. 10.1016/j.soilbio.2021.108472

[23] Poretsky R, Rodriguez-R LM, Luo CW et al. Strengths and limitations of 16S rRNA gene amplicon sequencing in revealing temporal microbial community dynamics. *PLoS One* 2014;9:e93827. 10.1371/journal.pone.0093827 24714158 PMC3979728

[24] Li ZM, Xia JJ, Jiang LYQ et al. Characterization of the human skin resistome and identification of two microbiota cutotypes. *Microbiome* 2021;9:47. 10.1186/s40168-020-00995-7 33597039 PMC7890624

[25] Liu XB, Burras CL, Kravchenko YS et al. Overview of Mollisols in the world: distribution, land use and management. *Can J Soil Sci* 2012;92:383–402. 10.4141/cjss2010-058

[26] Rui YC, Jackson RD, Cotrufo MF et al. Persistent soil carbon enhanced in Mollisols by well-managed grasslands but not annual grain or dairy forage cropping systems. *Proc Natl Acad Sci USA* 2022;119:e2118931119. 10.1073/pnas.2118931119 35145033 PMC8851490

[27] Shi JJ, Huang JF, Zhang F. Multi-year monitoring of paddy rice planting area in Northeast China using MODIS time series data. *Journal of Zhejiang University-Science B* 2013;14:934–46. 10.1631/jzus.B1200352 24101210 PMC3796645

[28] Sun XZ, Li TT, Zhang JZ. Soil health and microbial network analysis in a wheat-maize cropping system under different wheat yields. *Frontiers of Agricultural Science and Engineering* 2024;11:615–25. 10.15302/j-fase-2024570

[29] Nayab G, Zhou J, Jia R et al. Climate warming masks the negative effect of microplastics on plant-soil health in a silt loam soil. *Geoderma* 2022;425:116083. 10.1016/j.geoderma.2022.116083

[30] Chu JC, Zhou J, Wang Y et al. Field application of biodegradable microplastics has no significant effect on plant and soil health in the short term. *Environ Pollut* 2023;316:120556. 10.1016/j.envpol.2022.120556 36328286

[31] Wu QY, Congreves KA. A soil health scoring framework for arable cropping systems in Saskatchewan, Canada. *Canadian Journal of Soil Science* 2022;102:341–58. 10.1139/cjss-2021-0045

[32] Bi CJ, Chen ZL, Wang J et al. Quantitative assessment of soil health under different planting patterns and soil types. *Pedosphere* 2013;23:194–204. 10.1016/s1002-0160(13)60007-7

[33] Chen YX, Chen YS, Shi CM et al. SOAPnuke: a MapReduce acceleration-supported software for integrated quality control and preprocessing of high-throughput sequencing data. *GigaScience* 2018;7:1–6. 10.1093/gigascience/gix120 PMC5788068 29220494

[34] Buchfink B, Reuter K, Drost HG. Sensitive protein alignments at tree-of-life scale using DIAMOND. *Nat Methods* 2021;18:366–8. 10.1038/s41592-021-01101-x 33828273 PMC8026399

[35] Kanehisa M, Goto S, Sato Y et al. Data, information, knowledge and principle: back to metabolism in KEGG. *Nucleic Acids Res* 2014;42:D199–205. 10.1093/nar/gkt1076 24214961 PMC3965122

[36] Dang F, Wang QY, Yan XL et al. Threats to terrestrial plants from emerging nanoplastics. *ACS Nano* 2022;16:17157–67. 10.1021/acsnano.2c07627 36200753

[37] Jia YY, Hu XG, Kang WL et al. Unveiling microbial nitrogen metabolism in rivers using a machine learning approach. *Environ Sci Technol* 2024;58:6605–15. 10.1021/acs.est.3c09653 38566483

[38] Palansooriya KN, Li J, Dissanayake PD et al. Prediction of soil heavy metal immobilization by biochar using machine learning. *Environ Sci Technol* 2022;56:4187–98. 10.1021/acs.est.1c08302 35289167 PMC8988308

[39] Wijaya J, Byeon H, Jung WS et al. Machine learning modeling using microbiome data reveal microbial indicator for oil-contaminated groundwater. *J Water Process Eng* 2023;53:103610. 10.1016/j.jwpe.2023.103610

[40] Ji L, Tian CJ, Kuramae EE. Phosphorus-mediated succession of microbial nitrogen, carbon, and sulfur functions in rice-driven saline-alkali soil remediation. *Soil Biol Biochem* 2023;184:109125. 10.1016/j.soilbio.2023.109125

[41] Gu HD, Hu XJ, Zhang JY et al. Biogeographic patterns of viral communities, ARG profiles and virus-ARG associations in adjacent paddy and upland soils across black soil region. *J Hazard Mater* 2025;485:136909. 10.1016/j.jhazmat.2024.136909 39700951

[42] Oksanen J, Blanchet FG, Kindt R et al. *Package ‘vegan’* 2013;2:1–295.

[43] R Core Team . R: A Language and Environment for Statistical Computing. R Foundation for Statistical Computing, Vienna, Austria. 2021. https://www.R-project.org/.

[44] Ding LJ, Zhou XY, Zhu YG. Microbiome and antibiotic resistome in household dust from Beijing, China. *Environ Int* 2020;139:105702. 10.1016/j.envint.2020.105702 32248025

[45] Moebius-Clune BN, Moebius-Clune DJ, Gugino BK et al. Comprehensive Assessment of Soil Health—the Cornell Framework, 3rd edn. Geneva, NY: Cornell University, 2016.

[46] Wang L, Seki K, Miyazaki T et al. The causes of soil alkalinization in the Songnen plain of Northeast China. *Paddy Water Environ* 2009;7:259–70. 10.1007/s10333-009-0166-x

[47] Yang JC, Zhang SW, Li Y et al. Dynamics of saline-alkali land and its ecological regionalization in Western Songnen plain, China. *Chin Geogr Sci* 2010;20:159–66. 10.1007/s11769-010-0159-0

[48] Mahajan GR, Das B, Morajkar S et al. Comparison of soil quality indexing methods for salt-affected soils of Indian coastal region. *Environ Earth Sci* 2021;80:725. 10.1007/s12665-021-09922-x

[49] Bidalia A, Vikram K, Yamal G et al. Effect of salinity on soil nutrients and plant health. In: Akhtar M. (ed.), Salt Stress, Microbes, and Plant Interactions: Causes and Solution, Vol. 1. Singapore: Springer, 2019, 273–97.

[50] Lv BS, Li XW, Ma HY et al. Differences in growth and physiology of rice in response to different saline-alkaline stress factors. *Agron J* 2013;105:1119–28. 10.2134/agronj2013.0017er

[51] Jiao F, Shi XR, Han FP et al. Increasing aridity, temperature and soil pH induce soil C-N-P imbalance in grasslands. *Sci Rep* 2016;6:19601. 10.1038/srep19601 26792069 PMC4726211

[52] Wang MC, Liu XN, Liu ZW et al. Evaluation and driving force analysis of cultivated land quality in black soil region of Northeast China. *Chin Geogr Sci* 2023;33:601–15. 10.1007/s11769-023-1361-1

[53] Gu ZJ, Xie Y, Gao Y et al. Quantitative assessment of soil productivity and predicted impacts of water erosion in the black soil region of northeastern China. *Sci Total Environ* 2018;637-638:706–16. 10.1016/j.scitotenv.2018.05.061 29758427

[54] Chen KH, Feng J, Bodelier PLE et al. Metabolic coupling between soil aerobic methanotrophs and denitrifiers in rice paddy fields. *Nat Commun* 2024;15:3471. 10.1038/s41467-024-47827-y 38658559 PMC11043409

[55] Abid AA, Yu SH, Zou X et al. Unraveling nitrogen loss in paddy soils: a study of anaerobic nitrogen transformation in response to various irrigation practice. *Environ Res* 2024;252:118693. 10.1016/j.envres.2024.118693 38537742

[56] Lan T, Han Y, Cai ZC. Denitrification and its product composition in typical Chinese paddy soils. *Biol Fertil Soils* 2015;51:89–98. 10.1007/s00374-014-0953-4

[57] Kanekar PP, Kanekar SP. Halophilic and halotolerant microorganisms. In: Kanekar P.P., Kanekar S.P. (eds.), Diversity and Biotechnology of Extremophilic Microorganisms from India. Singapore: Springer Nature, 2022, 13–69.

[58] Zhang DC, Liu HC, Xin YH et al. *Planomicrobium glaciei* sp nov., a psychrotolerant bacterium isolated from a glacier. *Int J Syst Evol Microbiol* 2009;59:1387–90. 10.1099/ijs.0.002592-0 19502321

[59] Gan LZ, Zhang Y, Zhang LL et al. *Planococcus halotolerans* sp nov., isolated from a saline soil sample in China. *Int J Syst Evol Microbiol* 2018;68:3500–5. 10.1099/ijsem.0.003019 30265231

[60] Huang LY, Liu X, Tang JH et al. Electrochemical evidence for direct interspecies electron transfer between *Geobacter sulfurreducens* and *Prosthecochloris aestuarii*. *Bioelectrochemistry* 2019;127:21–5. 10.1016/j.bioelechem.2019.01.002 30641310

[61] Oechaiyaphum K, Ullah H, Shrestha RP et al. Impact of long-term agricultural management practices on soil organic carbon and soil fertility of paddy fields in Northeastern Thailand. *Geoderma Reg* 2020;22:e00307. 10.1016/j.geodrs.2020.e00307

[62] Wang XM, Wu M, Wei ZJ et al. Investigating drivers of free-living diazotroph activity in paddy soils across China. *Soil Biol Biochem* 2024;199:109601. 10.1016/j.soilbio.2024.109601

[63] Chaintreuil C, Giraud E, Prin Y et al. Photosynthetic bradyrhizobia are natural endophytes of the African wild rice *Oryza breviligulata*. *Appl Environ Microbiol* 2000;66:5437–47. 10.1128/aem.66.12.5437-5447.2000 11097925 PMC92479

[64] Piromyou P, Greetatorn T, Teamtisong K et al. Preferential association of endophytic Bradyrhizobia with different rice cultivars and its implications for rice endophyte evolution. *Appl Environ Microbiol* 2015;81:3049–61. 10.1128/aem.04253-14 25710371 PMC4393458

[65] Pandey A, Suter H, He JZ et al. Dissimilatory nitrate ammonification and N_2_ fixation helps maintain nitrogen nutrition in resource-limited rice paddies. *Biol Fertil Soils* 2021;57:107–15. 10.1007/s00374-020-01508-2

[66] Ding ZX, Wen T, Teng XY et al. Enhancing soil citrulline degrading function to mitigate soil-borne Fusarium wilt. *Nat Commun* 2026;17:1868. 10.1038/s41467-026-68606-x 41559060 PMC12923898

[67] Iqbal S, Ullah N, Janjua HA. *In vitro* evaluation and genome mining of *Bacillus subtilis* strain RS10 reveals its biocontrol and plant growth-promoting potential. *Agriculture* 2021;11:1273. 10.3390/agriculture11121273

[68] Zhang ZC, Sun JY, Wang D et al. Effects of rotation corn on potato yield, quality, and soil microbial communities. *Front Microbiol* 2025;16:1493333. 10.3389/fmicb.2025.1493333 40309109 PMC12040919

[69] Liao XH, Zhao J, Yi Q et al. Metagenomic insights into the effects of organic and inorganic agricultural managements on soil phosphorus cycling. *Agric Ecosyst Environ* 2023;343:108281. 10.1016/j.agee.2022.108281

[70] Ding LJ, Su JQ, Li H et al. Bacterial succession along a long-term chronosequence of paddy soil in the Yangtze River Delta, China. *Soil Biol Biochem* 2017;104:59–67. 10.1016/j.soilbio.2016.10.013

[71] Liang X, Zhu Y, Liu HY et al. Nitrogen-fixing cyanobacteria enhance microbial carbon utilization by modulating the microbial community composition in paddy soils of the Mollisols region. *Sci Total Environ* 2024;929:172609. 10.1016/j.scitotenv.2024.172609 38663623

[72] Wang ZM, Wang ZY, Zhang ZD et al. Comparative transcriptome reveals lignin biosynthesis being the key molecular pathway regulating oilseed rape growth treated by SiO_2_ NPs and biochar. *J Plant Res* 2025;138:147–59. 10.1007/s10265-024-01590-9 39537940

[73] Huang SY, Lentendu G, Fujinuma J et al. Soil micro-eukaryotic diversity patterns along elevation gradient are best estimated by increasing the number of elevation steps rather than within elevation band replication. *Microb Ecol* 2023;86:2606–17. 10.1007/s00248-023-02259-x 37458790 PMC10640418

[74] Dai ZM, Guo X, Lin JH et al. Metallic micronutrients are associated with the structure and function of the soil microbiome. *Nat Commun* 2023;14:8456. 10.1038/s41467-023-44182-2 38114499 PMC10730613

[75] Sveen TR, Viketoft M, Bengtsson J et al. Functional diversity of soil microbial communities increases with ecosystem development. *Nat Commun* 2025;16:10408. 10.1038/s41467-025-66544-8 41274910 PMC12644890

[76] Duan YL, Zhang JB, Petropoulos E et al. Soil acidification destabilizes terrestrial ecosystems via decoupling soil microbiome. *Glob Chang Biol* 2025;31:e70174. 10.1111/gcb.70174 40183155

