# Supplementary material for: Unveiling the role of soil microorganisms in indicating paddy soil health via metagenomics combined with machine learning
Source: ISME Commun. 2026 May 15;6(1):ycag133. doi: 10.1093/ismeco/ycag133 (PMC13271413; doi:10.1093/ismeco/ycag133)
Supplement: Supplemental_Material_ycag133 [file supplemental_material_ycag133.docx]

**Supplementary** **Material for**

**Unveiling the role of soil microorganisms in indicating paddy soil health via metagenomics combined with machine learning**

Yu-Ling Zheng^1,2^, Yun-Shuo Guo^1,2^, Xin-Yue Ren^2,3^, Yi-Fei Wang^3,4^, Hui-Ling Cui^1,2^, Li-Mei Zhang^1,2^, Long-Jun Ding^1,2*^, Yong-Guan Zhu^1,2,3^

^1^ State Key Laboratory of Regional and Urban Ecology, Research Center for Eco-Environmental Sciences, Chinese Academy of Sciences, Beijing 100085, China.

^2^ University of Chinese Academy of Sciences, Beijing 100049, China.

^3^ State Key Laboratory of Regional and Urban Ecology, Institute of Urban Environment, Chinese Academy of Sciences, Xiamen 361021, China.

^4^ Zhejiang Key Laboratory of Pollution Control for Port-Petrochemical Industry, CAS Haixi Industrial Technology Innovation Center in Beilun, Ningbo 315830, China.

*Corresponding author:

**Long-Jun Ding**, State Key Laboratory of Regional and Urban Ecology, Research Center for Eco-Environmental Sciences, Chinese Academy of Sciences, No. 18 Shuangqing Road, Haidian District, Beijing 100085, China.

Email: ljding@rcees.ac.cn, Tel: +86-10-62849328, Fax: + 86-10-62936940.

This SI file contains a total of 23 pages including Supplementary Materials and Methods, Supplementary References, 7 Figures, and 6 Tables.

**Supplementary Materials and Methods**

*Site description and soil sampling*

Soil samples were collected from flooded paddy fields in the black soil region of Northeast China, covering 514,877 km^2^ (39.87–49.17°N; 121.68–134.48°E). The region of paddy soil sampling is of vital importance in rice production in China [1], and has a temperate monsoon climate with a pronounced seasonality. It is warm and rainy in summer while cold and dry in winter, with a mean annual temperature (MAT) of 1°C–10°C and a mean annual precipitation (MAP) being 386-932 mm. These MAT and MAP data were obtained from the National Tibetan Plateau Data Center (http://data.tpdc.ac.cn), and averaged for the years from 2000 to 2020. The soil samples were mainly classified as Phaeozems and Chernozems according to Harmonized World Soil Database v 1.2 (http://www.fao.org/land-water/databases-and-software/hwsd/en/).

All soil samples were separated into three subsamples: one was stored at –80℃ for soil DNA extraction; another was stored at 4℃ for analyses of soil enzyme activities [including β-glucosidase (BG), urease and phosphatase (PHOS)], ammonium nitrogen (NH_4_^+^-N), nitrate nitrogen (NO_3_^−^-N), dissolved organic carbon (DOC), and dissolved total nitrogen (DTN); and the remaining subsample was air-dried for determination of other soil properties such as pH, electrical conductivity (EC), soil organic matter (SOM), total nitrogen (TN), available phosphorus (AP), and available potassium (AK).

*Analytical methods of soil properties*

Soil pH was measured in a 1:2.5 soil:water (w/v) suspension with a pH meter (FE28, Mettler Toledo, Switzerland). Soil electrical conductivity (EC) was measured by a conductometer (ORION STARA212, Thermo Scientific, USA) in a 1:5 soil:water (w/v). Soil organic matter (SOM) was determined using the K_2_Cr_2_O_7_ oxidation approach [2]. Soil total nitrogen (TN) was determined by the Element Analyzer (Vario EL cube, Elementar, Germany) [3]. Soil dissolved organic carbon (DOC) and dissolved total nitrogen (DTN) were extracted with 0.5 M K_2_SO_4_ at a ratio of 1:5 (w/v) and determined by a total organic carbon/nitrogen analyzer (Multi N/C 3100, Analytikjena, Germany) [4]. Soil ammonium nitrogen (NH_4_^+^-N) and nitrate nitrogen (NO_3_^−^-N) were extracted with 2 M KCl at a ratio of 1:5 (w/v) and measured by a continuous flow analyzer (AA3, Seal, Norderstedt, Germany) [5]. For soil available phosphorus (AP), the neutral and alkaline soils were extracted with 0.5 M NaHCO_3_ (pH 8.5) at a ratio of 1:20 (w/v) and then determined by the molybdenum antimony blue colorimetric method, while the acid soils were extracted with ammonium fluoride-hydrochloric acid at a ratio of 1:10 (w/v) and then determined using the same colorimetric method as the neutral and alkaline soils [2]. Soil available potassium (AK) was extracted with 1 M CH_3_COONH_4_ (pH 7.0) at a ratio of 1:10 (w/v), and measured by the atomic absorption spectrometry [2].

Soil β-glucosidase (BG) and phosphatase (PHOS) activities were both represented by 4-methylumbelliferone released during incubation with buffered solution and measured using the fluorescence method, with the difference that buffered 4-methylumbelliferyl β-D-glucoside and 4-methylumbelliferyl phosphate were used to determine BG and PHOS, respectively [6, 7]. Soil urease activity was determined following the instructions of Soil Urease (S-UE) Activity Assay Kit (Solarbio Science & Technology Co., Ltd, Beijing, China).

*Soil health level classification standard*

We referred to the reported classification method and combined the original 5 classification levels of soil health index (SHI) into 3 levels [8]. We combined "very poor" and "poor" into a low health level, while "good" and "very good" into a high health level. The "medium" level as described was still retained as the medium health level in this study. Specifically, 0%-50%, 50%-65%, and 65%-100% of the maximum SHI were defined as the low, medium and high health levels, respectively. The specific grading standards were shown in Table S2.

*Metagenomic sequencing data processing*

All raw data were filtered using the SOAPnuke (v1.5.2) tool [9], which produced an average of 16 GB of clean data per sample. The MEGAHIT (v1.2.9) tool was then used to assemble these clean data into contigs [10], and the contigs with length ≥ 500 bp were selected as the final assembling result. The Prodigal (v2.6.3) tool was used to predict open reading frames (ORFs) from the assembled contigs [11]. The predicted gene sequences with 95% sequence identity and 90% coverage were clustered by the CD-HIT (v4.8.1) tool [12], and the longest gene of each cluster was chosen as the representative sequence to construct a non-redundant gene catalog. The clean data were mapped to the unigenes by the bowtie2 (v2.5.4) tool to calculate the number of matching reads [13], and transformed to transcripts per million (TPM) values. For taxonomic annotation of bacteria, archaea, fungi, protists, and virus, the DIAMOND (v2.1.11) tool was used to compare the non-redundant gene set against the National Center for Biotechnology Information non-redundant (NCBI-nr) database [14]. The functional annotation was performed using the DIAMOND (v2.1.11) tool on the Kyoto Encyclopedia of Genes and Genomes (KEGG) database [15].

*Machine learning (ML) model development*

The dataset comprising the relative species-level metagenomic abundance profiles and the SHI value was divided into training (80%) and testing (20%) sets using train_test_split. Hyperparameters were optimised via 10-fold cross-validation with the training set using the GridSearchCV function to prevent overfitting [16]. Different hyperparameters were included in the Random Forest (RF), Support Vector Regression (SVR), and eXtreme Gradient Boosting (XGBoost) algorithms during the tuning process. For the RF, the number of trees (n_estimators), maximum depth of the trees (max_depth), minimum number of samples required to split an internal node (min_samples_split), minimum number of samples required to be at a leaf node (min_samples_leaf), and number of features to consider for the best split (max_features) were the tuned hyperparameters [17]. For the SVR, the penalty parameter (*C*) and epsilon (*ε*) were the tuned hyperparameters [18]. For the XGBoost, number of gradient boosted trees (n_estimators), boosting learning rate (learning_rate), and maximum tree depth for base learners (max_depth) were the tuned hyperparameters [16]. The optimal ML model from the RF, SVR, and XGBoost algorithms was obtained after hyperparameter tuning. The optimal hyperparameters for these ML models were summarized in Table S3. The testing set was used for ML model validation. The model performance was evaluated by coefficient of determination (*R*^2^), root mean square error (*RMSE*), and mean absolute error (*MAE*) [19]. *R*^2^ represents the proportion of the variance in the predicted values that can be explained by the variance in the measured values. *RMSE* and *MAE* were used to evaluate the extent of deviations between the predicted values and the measured values. The equations for calculating these model performance indexes were as follows:

$R^{2}=1-\frac{\sum_{i=1}^{n} {(y-y^{'})}^{2}}{\sum_{i=1}^{n} {(y-\bar{y})}^{2}}$ Equation (S1)

$RMSE=\sqrt{\frac{\sum_{i=1}^{n} {(y-y^{'})}^{2}}{n}}$ Equation (S2)

$MAE=\frac{\sum_{i=1}^{n} \left| y-y^{'} \right|}{n}$ Equation (S3)

where *n* is the number of samples in the training or testing set, *y'* is the predicted value, *y* is the measured value, and *ȳ* is the average value of the measured value.

**Supplementary Figures**


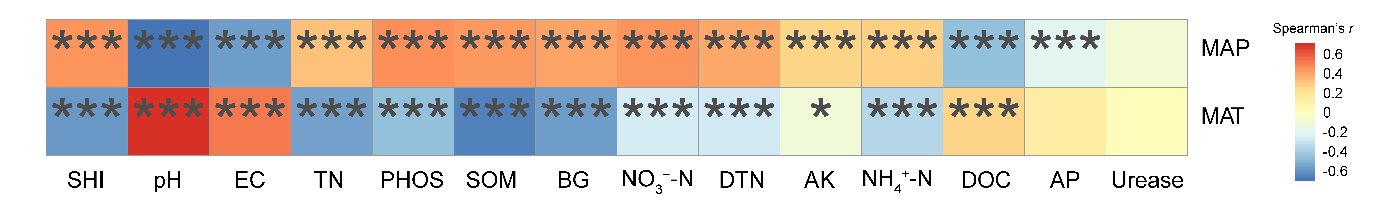


**Figure S1**. Heatmap plot of Spearman correlation analysis between soil health index (SHI)/soil properties and climatic factors [mean annual precipitation (MAP) and mean annual temperature (MAT)]. The color denotes the Spearman’s correlation coefficient. Significance level was set as *** *p* < 0.001, ** *p* < 0.01, and * *p* < 0.05. EC: electrical conductivity; TN: total nitrogen; PHOS: phosphatase; SOM: soil organic matter; BG: β-glucosidase; NO_3_^−^-N: nitrate nitrogen; DTN: dissolved total nitrogen; AK: available potassium; NH_4_^+^-N: ammonium nitrogen; DOC: dissolved organic carbon; and AP: available phosphorus.





**Figure S2.** (A) Relative abundances of the top 10 abundant microbial taxa at the genus level across the paddy soil samples. Genus was colored by its phylum. (B) Relative abundances of the top 10 KEGG pathways at level 3 across the paddy soil samples. KEGG pathway at level 3 was colored by KEGG pathway at level 1. KEGG: Kyoto Encyclopedia of Genes and Genomes.


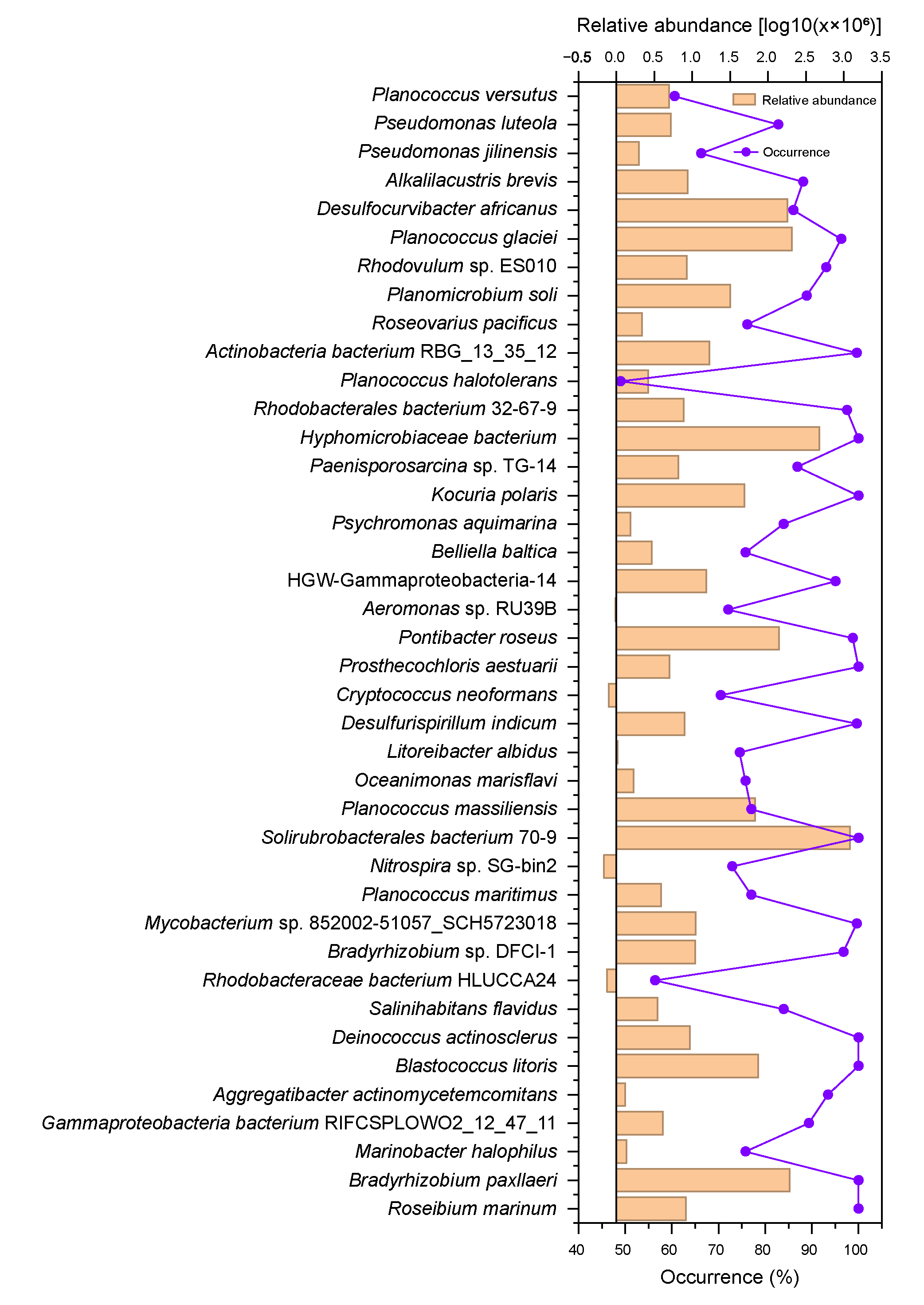


**Figure S3.** Mean relative abundance and occurrence of the 40 potential indicator taxa of paddy soil health.


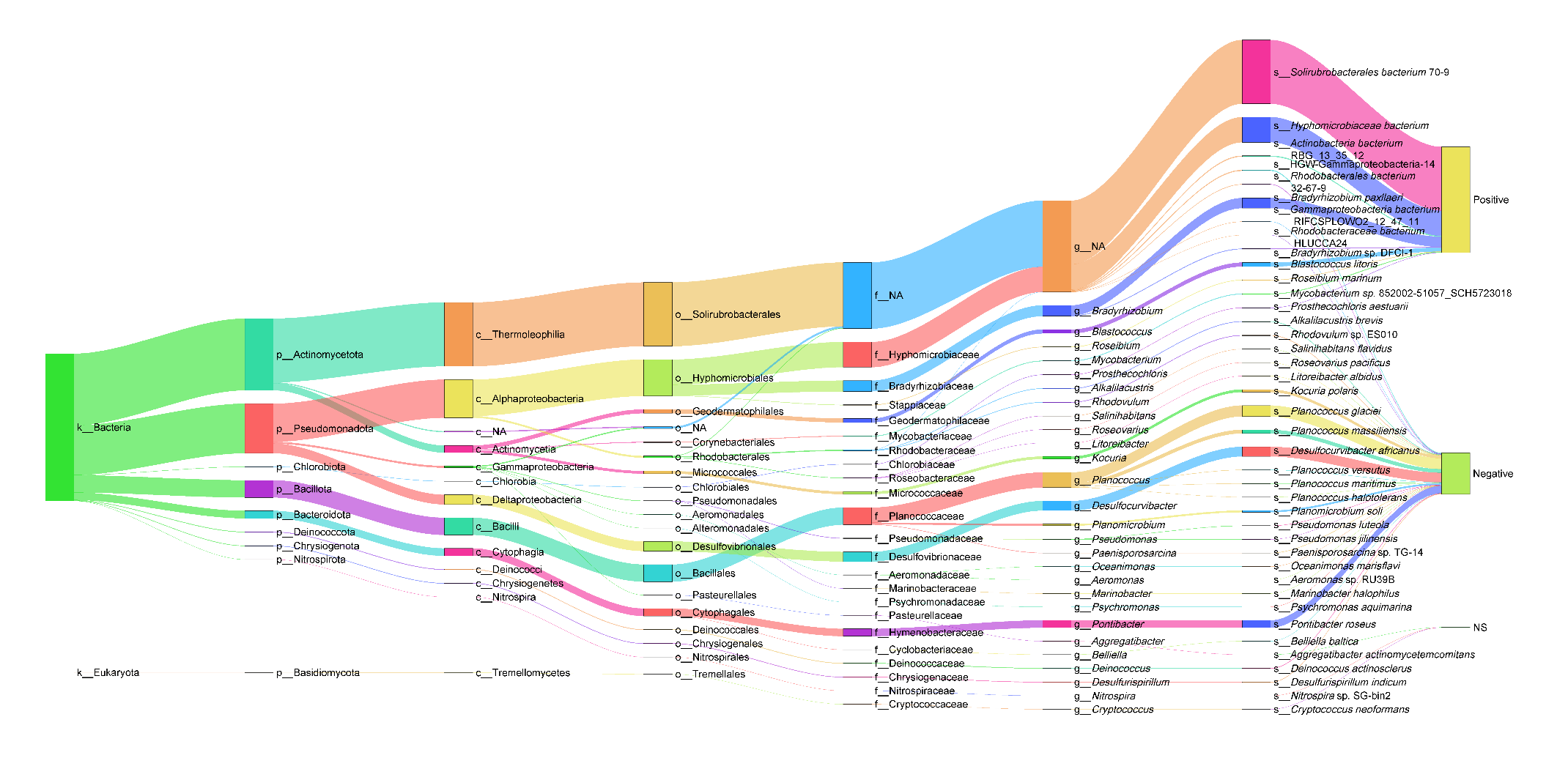


**Figure S4.** Taxonomic affiliations of the 40 potential indicator taxa of paddy soil health. ‘Positive’ and ‘Negative’ indicate that the microbial taxa are significantly and positively or negatively correlated with SHI according to Spearman correlation analysis (*p* < 0.05), while ‘NS’ denotes non-significant correlation (*p* ≥ 0.05). The name of microbial taxon with ‘NA’ indicates that the taxon cannot be assigned at the corresponding taxonomic level.


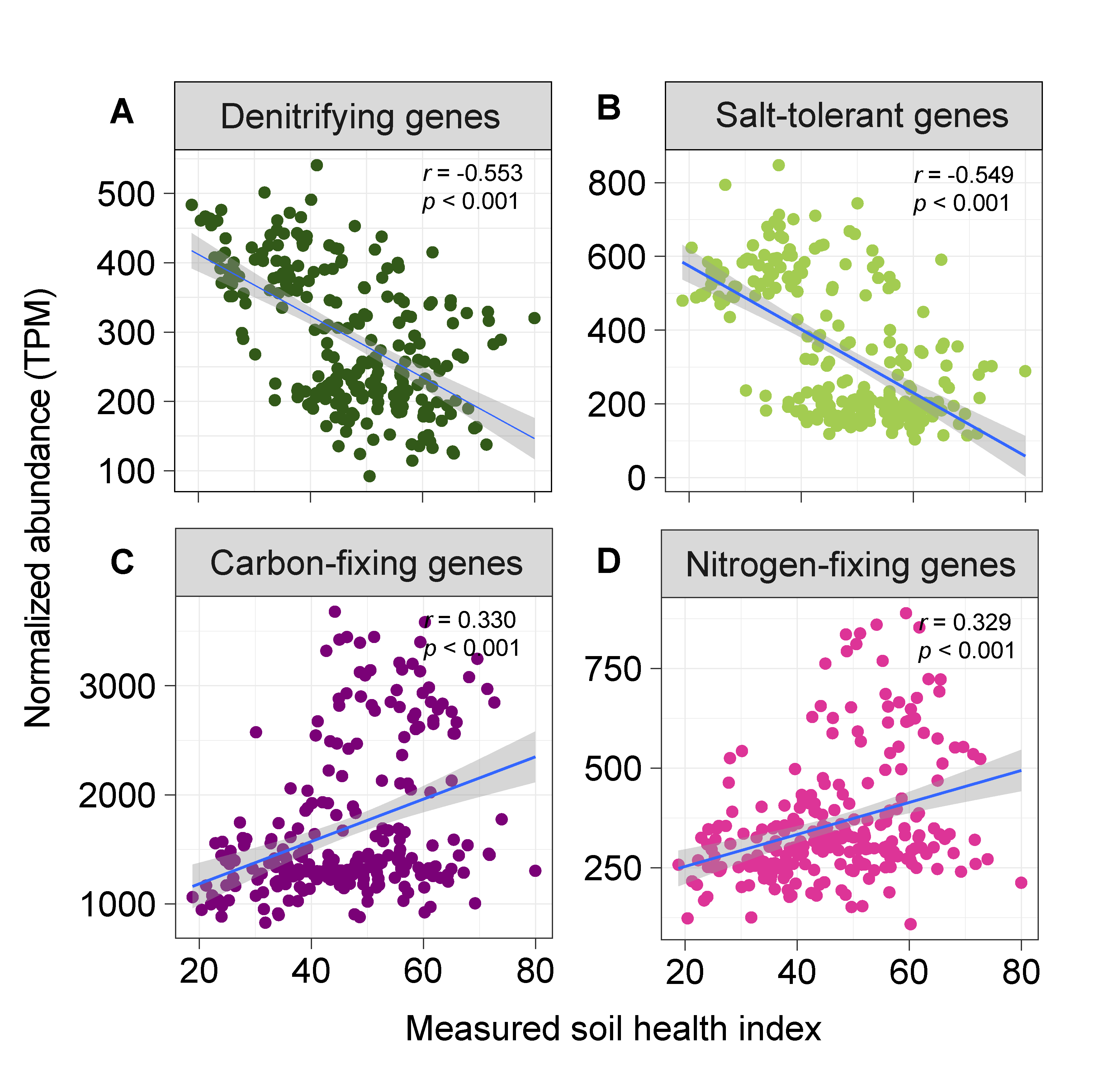


**Figure S5.** Relationships between the measured soil health index and the normalized abundances (indicated by TPM, transcripts per million) of (A) denitrifying genes, (B) salt-tolerant genes, (C) carbon-fixing genes, and (D) nitrogen-fixing genes.


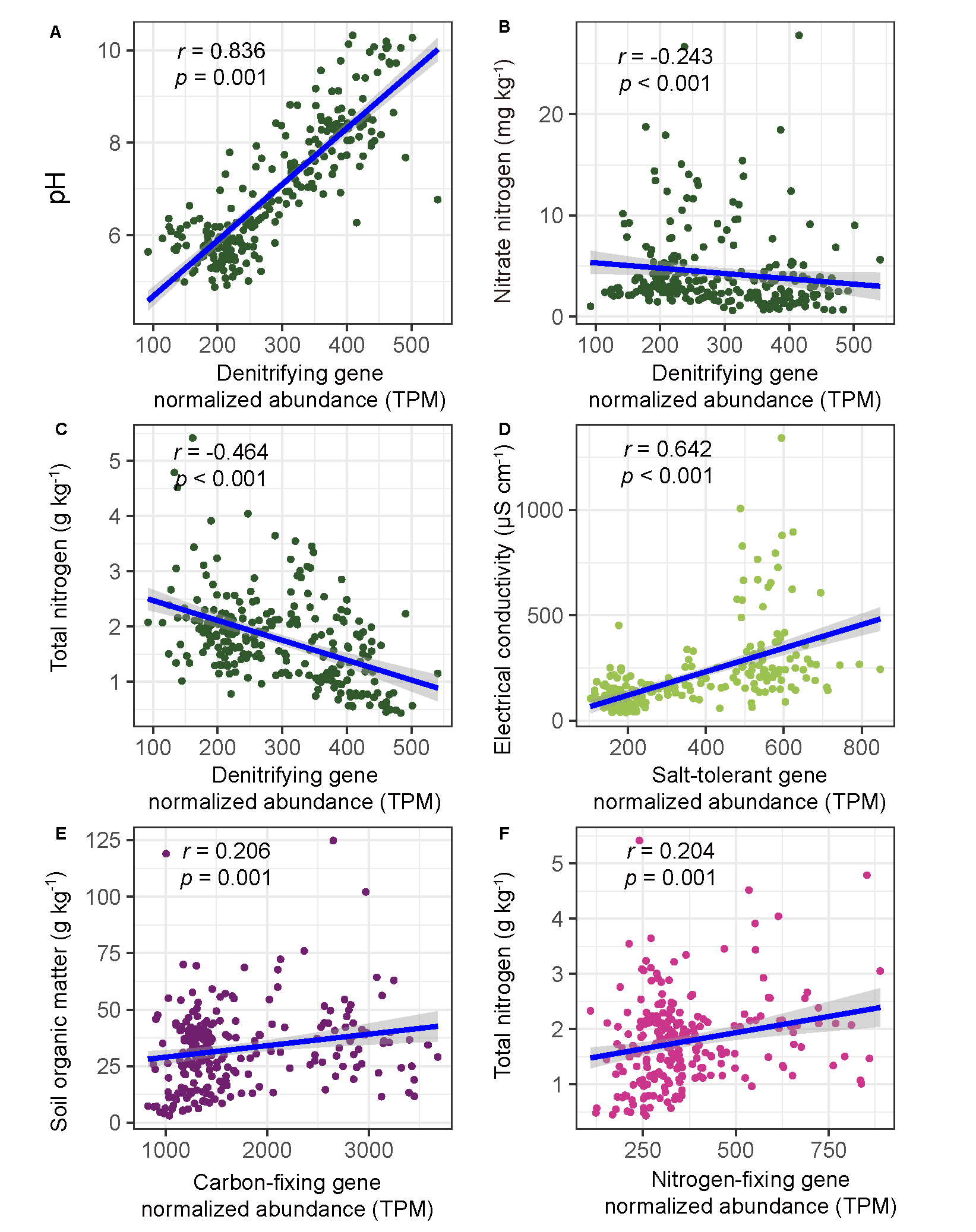


**Figure S6.** Relationships between soil properties and the normalized abundances (indicated by TPM, transcripts per million) of (A-C) denitrifying genes, (D) salt-tolerant genes, (E) carbon-fixing genes, and (F) nitrogen-fixing genes.


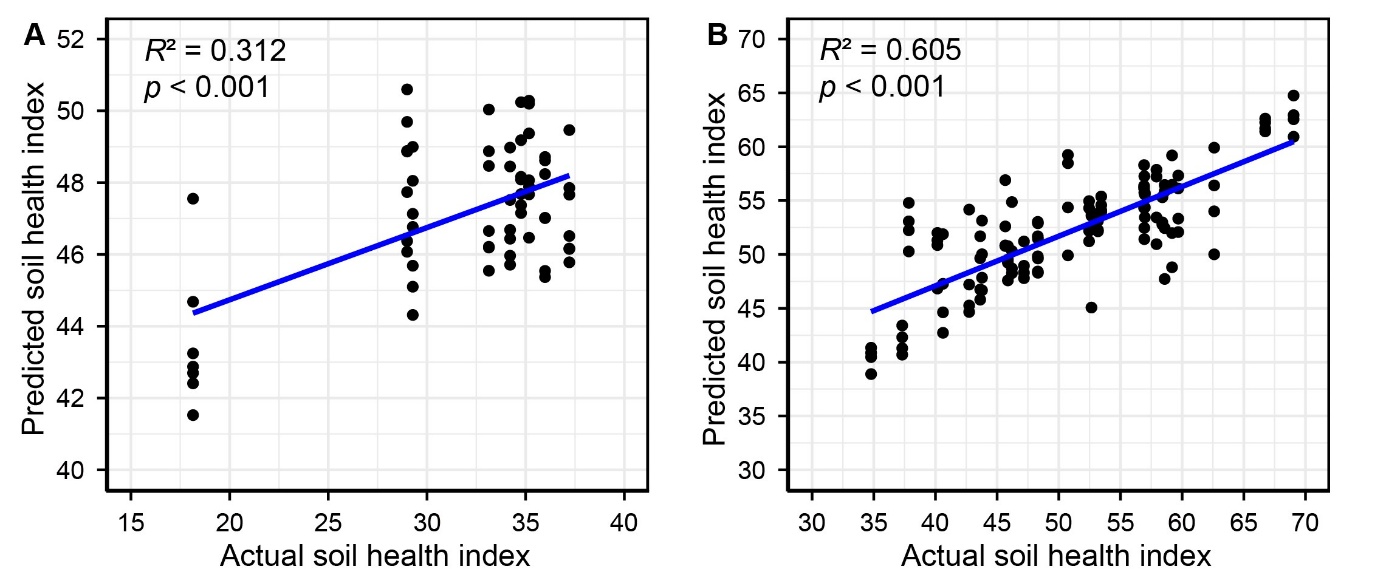


**Figure S7.** Relationships between predicted and actual soil health index (A) in bare soil and a chronosequence of paddy soils, and (B) across the typical Mollisol region of Northeast China.

**Supplementary Tables**

**Table S1.** Soil properties and climatic factors [mean annual precipitation (MAP) and mean annual temperature (MAT))] of the 243 tested paddy soils in Northeast China (See Table S1 in Excel).

**Table S2.** Grading standards of soil health levels based on soil health index (SHI) for the examined paddy soils.

| Discrepant percent based on the maximum SHI | SHI range | Soil health level |
| --- | --- | --- |
| 50% | SHI < 40 | Low |
| 65% | 40 ≤ SHI < 52 | Medium |
| 100% | 52 ≤ SHI | High |

**Table S3.** Prediction results of three machine learning (ML) models including Random Forest (RF), Support Vector Regression (SVR), and eXtreme Gradient Boosting (XGBoost).

| ML model | *R*^2^_Val_ | *RMSE*_Val_ | *MAE*_Val_ | *R*^2^_Tra_ | *RMSE*_Tra_ | *MAE*_Tra_ | *R*^2^_Test_ | *RMSE*_Test_ | *MAE*_Test_ | Hyper-parameter |
| --- | --- | --- | --- | --- | --- | --- | --- | --- | --- | --- |
| RF | 0.55 ± 0.17 | 7.87 ± 1.69 | 6.40 ± 1.31 | 0.94 | 3.04 | 2.39 | 0.65 | 7.71 | 6.23 | {'max_depth': 20, 'max_features': None, 'min_samples_leaf': 1, 'min_samples_split': 2, 'n_estimators': 200} |
| SVR | 0.57 ± 0.14 | 7.88 ± 2.08 | 6.19 ± 1.54 | 0.99 | 0.01 | 0.01 | 0.62 | 8.02 | 6.51 | {'C': 50, 'epsilon': 0.01} |
| XGBoost | 0.52 ± 0.22 | 8.03 ± 1.72 | 6.37 ± 1.48 | 0.99 | 0.02 | 0.004 | 0.60 | 8.21 | 6.60 | {'learning_rate': 0.05, 'max_depth': 10, 'n_estimators': 200} |

*R*^2^_Val_, *R*^2^_Tra_, and *R*^2^_Test_ represent the coefficients of determination (*R*^2^) between the measured and predicted soil health index for the cross-validation dataset, training dataset, and testing dataset, respectively.

*RMSE*_Val_, *RMSE*_Tra_, and *RMSE*_Test_ represent the root mean square error (*RMSE*) between the measured and predicted soil health index for the cross-validation dataset, training dataset, and testing dataset, respectively.

*MAE*_Val_, *MAE*_Tra_, and *MAE*_Test_ represent the mean absolute error (*MAE*) between the measured and predicted soil health index for the cross-validation dataset, training dataset, and testing dataset, respectively.

**Table S4.** The grouping of potential indicator taxa of paddy soil health identified in this study based on their potential functional roles.

| Importance order^1^ | Indicator taxa | Functional group | Reference |
| --- | --- | --- | --- |
| 1 | *Planococcus versutus* | Halophilic bacteria | [20] |
| 2 | *Pseudomonas luteola* | Denitrifying bacteria | [21] |
| 3 | *Pseudomonas jilinensis* | Denitrifying bacteria | [22] |
| 4 | *Alkalilacustris brevis* | Halophilic bacteria | [23] |
| 5 | *Desulfocurvibacter africanus* | Others^2^ | / |
| 6 | *Planococcus glaciei* | Halotolerant bacteria | [24] |
| 7 | *Rhodovulum* sp. ES010 | Halophilic bacteria | [25] |
| 8 | *Planomicrobium soli* | Halophilic bacteria | [26] |
| 9 | *Roseovarius pacificus* | Halophilic bacteria | [27] |
| 10 | *Actinobacteria bacterium* RBG_13_35_12 | Others | / |
| 11 | *Planococcus halotolerans* | Halotolerant bacteria | [28] |
| 12 | *Rhodobacterales bacterium* 32-67-9 | Others | / |
| 13 | *Hyphomicrobiaceae bacterium* | Others | [29] |
| 14 | *Paenisporosarcina* sp. TG-14 | Others | / |
| 15 | *Kocuria polaris* | Others | / |
| 16 | *Psychromonas aquimarina* | Halophilic bacteria | [30] |
| 17 | *Belliella baltica* | Denitrifying bacteria | [31] |
| 18 | HGW-Gammaproteobacteria-14 | Others | / |
| 19 | *Aeromonas* sp. RU39B | Others | / |
| 20 | *Pontibacter roseus* | Halotolerant bacteria | [32] |
| 21 | *Prosthecochloris aestuarii* | Carbon-fixing bacteria | [33] |
| 22 | *Cryptococcus neoformans* | Others | / |
| 23 | *Desulfurispirillum indicum* | Others | / |
| 24 | *Litoreibacter albidus* | Halophilic bacteria | [34] |
| 25 | *Oceanimonas marisflavi* | Others | / |
| 26 | *Planococcus massiliensis* | Halophilic bacteria | [35] |
| 27 | *Solirubrobacterales bacterium* 70-9 | Putative plant-beneficial bacteria^3^ | [36] |
| 28 | *Nitrospira* sp. SG-bin2 | Others | / |
| 29 | *Planococcus maritimus* | Halophilic bacteria | [37] |
| 30 | *Mycobacterium* sp. 852002-51057_SCH5723018 | Others | / |
| 31 | *Bradyrhizobium* sp. DFCI-1 | Nitrogen-fixing bacteria | [38] |
| 32 | *Rhodobacteraceae bacterium* HLUCCA24 | Others | / |
| 33 | *Salinihabitans flavidus* | Halophilic bacteria | [25] |
| 34 | *Deinococcus actinosclerus* | Others | / |
| 35 | *Blastococcus litoris* | Putative plant-beneficial bacteria | [39] |
| 36 | *Aggregatibacter actinomycetemcomitans* | Others | / |
| 37 | *Gammaproteobacteria bacterium* RIFCSPLOWO2_12_47_11 | Others | / |
| 38 | *Marinobacter halophilus* | Halophilic bacteria | [40] |
| 39 | *Bradyrhizobium paxllaeri* | Nitrogen-fixing bacteria | [38] |
| 40 | *Roseibium marinum* | Others | [41] |

^1^Importance order is determined based on SHAP value of the random forest model.

^2^“Others” refers to the potential indicator taxa whose ecological roles in soil health have not been reported till now, as well as those not significantly correlated with soil health index.

^3^‘Putative plant-beneficial bacteria’ refers to bacteria that have been reported to be capable of promoting plant growth, but their effects on soil health are unclear.

**Table S5.** List of functional genes associated with the potential indicator taxa of paddy soil health grouped by functional perspective.

| **Contig ID** | **Functional group** | **Indicator taxa** | **KEGG orthology number** | **Gene name** |
| --- | --- | --- | --- | --- |
| Contig_228010 | Denitrifying bacteria | *Pseudomonas luteola* | K02567 | *napA* |
| Contig_167478 | Halotolerant bacteria | *Planococcus glaciei* | K03316 | *nhaP* |
| Contig_578792 | Nitrogen-fixing bacteria | *Bradyrhizobium paxllaeri* | K02584 | *nifA* |
| Contig_383699 | Putative plant-beneficial bacteria | *Blastococcus litoris* | K01915 | *glnA* |
| Contig_353771 | Putative plant-beneficial bacteria | *Blastococcus litoris* | K01755 | *argH* |
| Contig_347904 | Putative plant-beneficial bacteria | *Solirubrobacterales bacterium* 70-9 | K01915 | *glnA* |
| Contig_624216 | Putative plant-beneficial bacteria | *Solirubrobacterales bacterium* 70-9 | K01755 | *argH* |
| Contig_137123 | Putative plant-beneficial bacteria | *Solirubrobacterales bacterium* 70-9 | K00135 | *gabD* |
| Contig_664614 | Putative plant-beneficial bacteria | *Solirubrobacterales bacterium* 70-9 | K01507 | *ppa* |
| Contig_204142 | Others | *Desulfocurvibacter africanus* | K00374 | *narI* |
| Contig_724461 | Others | *Kocuria polaris* | K00368 | *nirK* |
| Contig_519814 | Others | *Desulfurispirillum indicum* | K02567 | *napA* |
| Contig_27742 | Others | *Hyphomicrobiaceae bacterium* | K00830 | *AGXT* |

**Table S6.** The annotated functional genes associated with key ecological processes inferred from the functional grouping of the potential indicator taxa of paddy soil health identified in this study.

| **KEGG orthology number** | **Gene name** | **Encoded protein** |
| --- | --- | --- |
| **Denitrifying genes** |  |  |
| K00374 | *narI* | nitrate reductase gamma subunit |
| K02567 | *napA* | nitrate reductase (cytochrome) |
| K02568 | *napB* | nitrate reductase (cytochrome) |
| K00368 | *nirK* | nitrite reductase (NO-forming) |
| K15864 | *nirS* | nitrite reductase (NO-forming) |
| **Salt-tolerant genes** |  |  |
| K03313 | *nhaA* | Na^+/^H^+^ antiporter |
| K03316 | *nhaP* | Na^+^/H^+^ antiporter |
| K03498 | *trkH*, *trkG*, *ktrB*, *ktrD* | trk/ktr system potassium uptake protein |
| **Carbon-fixing genes** |  |  |
| K00031 | *icd* | isocitrate dehydrogenase |
| K00174 | *korA* | 2-oxoglutarate ferredoxin oxidoreductase subunit alpha |
| K00175 | *korB* | 2-oxoglutarate ferredoxin oxidoreductase subunit beta |
| **Nitrogen-fixing genes** |  |  |
| K02584 | *nifA* | Nif-specific regulatory protein |
| K02586 | *nifD* | nitrogenase molybdenum-iron protein alpha chain |
| K02588 | *nifH* | nitrogenase iron protein NifH |
| K02591 | *nifK* | nitrogenase molybdenum-iron protein beta chain |

**Supplementary References**

1. Xin FF, Xiao XM, Dong JW, Zhang GL, Zhang Y, Wu XC. *et al*. Large increases of paddy rice area, gross primary production, and grain production in Northeast China during 2000-2017. *Science of the Total Environment* 2020;**711**:135183. https://doi.org/10.1016/j.scitotenv.2019.135183

2. Lu RK. Analytical methods for soil and agro-chemistry. Beijing: China Agricultural Science and Technology Press, 2000.

3. Ding LJ, Su JQ, Sun GX, Wu JS, Wei WX. Increased microbial functional diversity under long-term organic and integrated fertilization in a paddy soil. *Applied Microbiology and Biotechnology* 2018;**102**:1969-1982. https://doi.org/10.1007/s00253-017-8704-8

4. de Sosa LL, Glanville HC, Marshall MR, Schnepf A, Cooper DM, Hill PW. *et al*. Stoichiometric constraints on the microbial processing of carbon with soil depth along a riparian hillslope. *Biology and Fertility of Soils* 2018;**54**:949-963. https://doi.org/10.1007/s00374-018-1317-2

5. Zhu GB, Wang SY, Wang Y, Wang CX, Risgaard-Petersen N, Jetten MSM. *et al*. Anaerobic ammonia oxidation in a fertilized paddy soil. *Isme Journal* 2011;**5**:1905-1912. https://doi.org/10.1038/ismej.2011.63

6. Saiya-Cork KR, Sinsabaugh RL, Zak DR. The effects of long term nitrogen deposition on extracellular enzyme activity in an *Acer saccharum* forest soil. *Soil Biology & Biochemistry* 2002;**34**:1309-1315. https://doi.org/10.1016/s0038-0717(02)00074-3

7. Bell CW, Fricks BE, Rocca JD, Steinweg JM, McMahon SK, Wallenstein MD. High-throughput fluorometric measurement of potential soil extracellular enzyme activities. *Jove-Journal of Visualized Experiments* 2013:**81**:e50961. https://doi.org/10.3791/50961

8. Bi CJ, Chen ZL, Wang J, Zhou D. Quantitative assessment of soil health under different planting patterns and soil types. *Pedosphere* 2013;**23**:194-204. https://doi.org/10.1016/s1002-0160(13)60007-7

9. Chen YX, Chen YS, Shi CM, Huang ZB, Zhang Y, Li SK. *et al*. SOAPnuke: a MapReduce acceleration-supported software for integrated quality control and preprocessing of high-throughput sequencing data. *GigaScience* 2018;**7**:1-6. https://doi.org/10.1093/gigascience/gix120

10. Li DH, Luo RB, Liu CM, Leung CM, Ting HF, Sadakane K. *et al*. MEGAHIT v1.0: A fast and scalable metagenome assembler driven by advanced methodologies and community practices. *Methods* 2016;**102**:3-11. https://doi.org/10.1016/j.ymeth.2016.02.020

11. Hyatt D, Chen GL, LoCascio PF, Land ML, Larimer FW, Hauser, LJ. Prodigal: prokaryotic gene recognition and translation initiation site identification. *BMC Bioinformatics* 2010;**11**:119. https://doi.org/10.1186/1471-2105-11-119

12. Fu LM, Niu BF, Zhu ZW, Wu ST, Li WZ. CD-HIT: accelerated for clustering the next-generation sequencing data. *Bioinformatics* 2012;**28**:3150-3152. https://doi.org/10.1093/bioinformatics/bts565

13. Langmead B, Salzberg SL. Fast gapped-read alignment with Bowtie 2. *Nature Methods* 2012;**9**:357-359. https://doi.org/10.1038/nmeth.1923

14. Buchfink B, Reuter K, Drost HG. Sensitive protein alignments at tree-of-life scale using DIAMOND. *Nature Methods* 2021;**18**:366-368. https://doi.org/10.1038/s41592-021-01101-x

15. Kanehisa M, Goto S, Sato Y, Kawashima M, Furumichi M, Tanabe M. Data, information, knowledge and principle: back to metabolism in KEGG. *Nucleic Acids Research* 2014;**42**:D199-D205. https://doi.org/10.1093/nar/gkt1076

16. Jia YY, Hu XG, Kang WL, Dong Xu. Unveiling microbial nitrogen metabolism in rivers using a machine learning approach. *Environmental Science & Technology* 2024;**58**:6605-6615. https://doi.org/10.1021/acs.est.3c09653

17. Liu ZT, Ma RA, Zhu D, Konstantinidis KT, Zhu YG, Zhang SY. Organic fertilization co-selects genetically linked antibiotic and metal(loid) resistance genes in global soil microbiome. *Nature Communications* 2024;**15**:5168. https://doi.org/10.1038/s41467-024-49165-5

18. Yi YB, Liu TC, Merder J, He C, Bao HY, Li PH. *et al*. Unraveling the linkages between molecular abundance and stable carbon isotope ratio in dissolved organic matter using machine learning. *Environmental Science & Technology* 2023;**57**:17900-17909. https://doi.org/10.1021/acs.est.3c00221

19. Palansooriya KN, Li J, Dissanayake PD, Suvarna M, Li LY, Yuan XZ. *et al*. Prediction of soil heavy metal immobilization by biochar using machine learning. *Environmental Science & Technology* 2022;**56**:4187-4198. https://doi.org/10.1021/acs.est.1c08302

20. See-Too WS, Ee R, Madhaiyan M, Kwon SW, Tan JY, Lim YL. *et al*. *Planococcus versutus* sp nov., isolated from soil. *International Journal of Systematic and Evolutionary Microbiology* 2017;**67**:944-950. https://doi.org/10.1099/ijsem.0.001721

21. Avsar C, Aras ES. Community structures and comparison of *nosZ* and 16S rRNA genes from culturable denitrifying bacteria. *Folia Microbiologica* 2020;**65**:497-510. https://doi.org/10.1007/s12223-019-00754-8

22. Wang JW, Cai M, Nie Y, Hu B, Yang Y, Wu XL. *Pseudomonas jilinensis* sp. nov., isolated from oil production water of Jilin oilfield in China. *Current Microbiology* 2020;**77**:688-694. https://doi.org/10.1007/s00284-019-01798-2

23. Zhang R, Ju Z, Han SB, Hou XJ, Yu YH, Zhang XQ. *et al*. *Alkalilacustris brevis* gen. nov., sp. nov., isolated from a soda lake. *International Journal of Systematic and Evolutionary Microbiology* 2019;**69**:1669-1675. https://doi.org/10.1099/ijsem.0.003373

24. Zhang DC, Liu HC, Xin YH, Yu Y, Zhou PJ, Zhou YG. *Planomicrobium glaciei* sp nov., a psychrotolerant bacterium isolated from a glacier. *International Journal of Systematic and Evolutionary Microbiology* 2009;**59**:1387-1390. https://doi.org/10.1099/ijs.0.002592-0

25. Rosenberg E, DeLong EF, Lory S, Stackebrandt E, Thompson F. The prokaryotes: alphaproteobacteria and betaproteobacteria. Berlin, Heidelberg: Spring, 2014.

26. Luo XN, Zhang JL, Li D, Xin YH, Xin D, Fan L. *Planomicrobium soli* sp nov., isolated from soil. *International Journal of Systematic and Evolutionary Microbiology* 2014;**64**:2700-2705. https://doi.org/10.1099/ijs.0.055426-0

27. Wang BJ, Tan TF, Shao ZZ. *Roseovarius pacificus* sp nov., isolated from deep-sea sediment. *International Journal of Systematic and Evolutionary Microbiology* 2009;**59**:1116-1121. https://doi.org/10.1099/ijs.0.002477-0

28. Gan LZ, Zhang Y, Zhang LL, Li XG, Wang ZK, He LL. *et al*. *Planococcus halotolerans* sp nov., isolated from a saline soil sample in China. *International Journal of Systematic and Evolutionary Microbiology* 2018;**68**:3500-3505. https://doi.org/10.1099/ijsem.0.003019

29. Ding LJ, Su JQ, Li H, Zhu YG, Cao ZH. Bacterial succession along a long-term chronosequence of paddy soil in the Yangtze River Delta, China. *Soil Biology & Biochemistry* 2017;**104**:59-67. https://doi.org/10.1016/j.soilbio.2016.10.013

30. Miyazaki M, Nogi Y, Fujiwara Y, Horikoshi K. *Psychromonas japonica* sp nov., *Psychromonas aquimarina* sp nov., *Psychromonas macrocephali* sp nov and *Psychromonas ossibalaenae* sp nov., psychrotrophic bacteria isolated from sediment adjacent to sperm whale carcasses off Kagoshima, Japan. *International Journal of Systematic and Evolutionary Microbiology* 2008;**58**:1709-1714. https://doi.org/10.1099/ijs.0.65744-0

31. Harter J, Weigold P, El-Hadidi M, Huson DH, Kappler A, Behrens S. Soil biochar amendment shapes the composition of N_2_O-reducing microbial communities. *Science of the Total Environment* 2016;**562**:379-390. https://doi.org/10.1016/j.scitotenv.2016.03.220

32. Mukherjee S, Lapidus A, Shapiro N, Cheng JF, Han J, Reddy TBK. *et al*. High quality draft genome sequence and analysis of *Pontibacter roseus* type strain SRC-1^T^ (DSM 17521^T^) isolated from muddy waters of a drainage system in Chandigarh, India. *Standards in Genomic Sciences* 2015;**10**:8. https://doi.org/10.1186/1944-3277-10-8

33. Huang LY, Liu X, Tang JH, Yu LP, Zhou SG. Electrochemical evidence for direct interspecies electron transfer between *Geobacter sulfurreducens* and *Prosthecochloris aestuarii*. *Bioelectrochemistry* 2019;**127**:21-25. https://doi.org/10.1016/j.bioelechem.2019.01.002

34. Romanenko LA, Tanaka N, Frolova GM, Svetashev VI, Mikhailov VV. *Litoreibacter albidus* gen. nov., sp. nov. and *Litoreibacter janthinus* sp. nov., members of the class *Alphaproteobacteria* isolated from the seashore. *International Journal of Systematic and Evolutionary Microbiology* 2011;**61**:148-154. https://doi.org/10.1099/ijs.0.019513-0

35. Seck EH, Sankar SA, Khelaifia S, Croce O, Robert C, Couderc C. *et al*. Noncontiguous finished genome sequence and description of *Planococcus massiliensis* sp. nov., a moderately halophilic bacterium isolated from the human gut*. New Microbes and New Infections* 2016;**10**:36-46. https://doi.org/10.1016/j.nmni.2015.12.006

36. Xu LT, Jin S, Su Y, Lyu XC, Yan SS, Wang C. *et al*. Combined metagenomics and metabolomic analysis of microbial community structure and metabolic function in continuous soybean cropping soils of Songnen Plain, China. *Chemical and Biological Technologies in Agriculture* 2024;**11**:46. https://doi.org/10.1186/s40538-024-00569-x

37. Waghmode S, Suryavanshi M, Dama L, Kansara S, Ghattargi V, Das P. *et al*. Genomic insights of halophilic *Planococcus maritimus* SAMP MCC 3013 and detail investigation of its biosurfactant production. *Frontiers in Microbiology* 2019;**10**:235. https://doi.org/10.3389/fmicb.2019.00235

38. Wang XM, Wu M, Wei ZJ, Hazard C, Nicol GW, Zhao HC. *et al*. Investigating drivers of free-living diazotroph activity in paddy soils across China. *Soil Biology & Biochemistry* 2024;**199**:109601. https://doi.org/10.1016/j.soilbio.2024.109601

39. Ma TF, He XH, Chen SG, Li YJ, Huang QW, Xue C. *et al*. Long-term organic-inorganic fertilization regimes alter bacterial and fungal communities and rice yields in paddy soil. *Frontiers in Microbiology* 2022;**13**:890712. https://doi.org/10.3389/fmicb.2022.890712

40. Zhong ZP, Liu Y, Liu HC, Wang F, Zhou YG, Liu ZP. *Marinobacter halophilus* sp nov., a halophilic bacterium isolated from a salt lake. *International Journal of Systematic and Evolutionary Microbiology* 2015;**65**:2838-2845. https://doi.org/10.1099/ijs.0.000338

41. Liang X, Zhu Y, Liu HY, Xie ZM, Li GB, Li DH. *et al*. Nitrogen-fixing cyanobacteria enhance microbial carbon utilization by modulating the microbial community composition in paddy soils of the Mollisols region. *Science of the Total Environment* 2024;**929**:172609. https://doi.org/10.1016/j.scitotenv.2024.172609
